# Structures of prokaryotic ubiquitin-like protein Pup in complex with depupylase Dop reveal the mechanism of catalytic phosphate formation

**DOI:** 10.1038/s41467-021-26848-x

**Published:** 2021-11-17

**Authors:** Hengjun Cui, Andreas U. Müller, Marc Leibundgut, Jiawen Tian, Nenad Ban, Eilika Weber-Ban

**Affiliations:** ETH Zurich, Institute of Molecular Biology & Biophysics, CH-8093 Zurich, Switzerland

**Keywords:** Enzyme mechanisms, Fluorescence spectroscopy, Post-translational modifications, X-ray crystallography

## Abstract

Pupylation is the post-translational modification of lysine side chains with prokaryotic ubiquitin-like protein (Pup) that targets proteins for proteasomal degradation in mycobacteria and other members of Actinobacteria. Pup ligase PafA and depupylase Dop are the two enzymes acting in this pathway. Although they share close structural and sequence homology indicative of a common evolutionary origin, they catalyze opposing reactions. Here, we report a series of high-resolution crystal structures of Dop in different functional states along the reaction pathway, including Pup-bound states in distinct conformations. In combination with biochemical analysis, the structures explain the role of the C-terminal residue of Pup in ATP hydrolysis, the process that generates the catalytic phosphate in the active site, and suggest a role for the Dop-loop as an allosteric sensor for Pup-binding and ATP cleavage.

## Abstract (web summary)

Pupylation is a bacterial post-translational protein modification, where the small ubiquitin-like protein Pup is covalently attached to lysine side chains of target proteins, which is a reversible process and depupylation is catalysed by the depupylase enzyme, Dop. Here, the authors present crystal structures of Dop in different functional states, which together with biochemical experiments provide insights into the catalytic mechanism of this enzyme.

## Introduction

Mycobacteria and other actinobacteria evolved a post-translational protein modification pathway termed pupylation that is functionally related to eukaryotic ubiquitination and can target proteins to a bacterial proteasome complex for degradation^1–3^. *Mycobacterium tuberculosis* (Mtb), one of the deadliest human pathogens to date, benefits from this pathway for its survival inside the host^4–6^. Protein quality control mechanisms and protein degradation pathways have recently garnered high interest as targets for the development of new drugs against increasingly drug-resistant Mtb strains that cause untreatable tuberculosis infections^7,8^. An understanding of the regulation of pupylation on the molecular level is therefore not only of fundamental scientific interest but also of potential medical relevance for drug development to interfere with this bacterium’s survival in the host.

During the process of pupylation, prokaryotic ubiquitin-like protein (Pup) is covalently attached via the γ-carboxylate of its C-terminal glutamate to a lysine side chain in the target protein by the formation of an isopeptide bond^9,10^. While the bacterial proteasome is homologous to the eukaryotic proteasome and was likely obtained by horizontal gene transfer^11,12^, the modification enzymes that catalyze pupylation and depupylation evolved from an ancient bacterial glutamine synthetase-like enzyme^13^. A single Pup ligase enzyme, PafA (proteasome accessory factor A), is responsible for modifying all pupylation substrate proteins^3,9^ and likewise a single depupylase enzyme, Dop (deamidase of Pup), removes Pup from Pup-protein adducts^10,14,15^. In some actinobacterial species including Mtb, where Pup is encoded with a C-terminal glutamine (PupQ), Dop also acts as deamidase to generate ligation-competent Pup with a C-terminal glutamate (PupE)^9^. The ligase and depupylase enzymes are close sequence and structural homologs, featuring a large N-terminal domain that contains the active site and is homologous to glutamine synthetase (GS) and other γ-glutamyl-amine ligases^16^. A small C-terminal domain unique to the pupylation enzymes lies adjacent to the active site and closes it off on one side^16^. The active sites of Dop and PafA feature a curved anti-parallel β-sheet cradle with nucleotide bound at one end of the cradle and the binding site for the C-terminal glutamate residue of Pup located at the other end^16,17^. The phosphate chain of the nucleotide runs along the β-strands of the cradle towards the glutamate^17^.

In PafA, ATP is required for activation of the side-chain carboxylate of Pup’s C-terminal glutamate by transferring the γ-phosphate to Pup, forming a mixed glutamyl-phosphate anhydride and turning over ATP to ADP in the process^18^. The carboxyl-carbon of the phospho-Pup intermediate is then attacked by the amino group of a lysine side-chain in the target protein to form the isopeptide bond^18^. ATP turnover in PafA is therefore part of the catalytic cycle and is stoichiometric with substrate turnover. Dop on the other hand does not turn over ATP stoichiometrically with substrate^9,19^. Rather, before the catalytic cycle of Dop can begin, Dop-mediated cleavage of ATP in the active site must take place to generate the Pi species important for catalysis^19^. The turnover of ATP is therefore stoichiometric with Dop active sites rather than substrate turnover, and ATP hydrolysis is not part of the depupylation/deamidation reaction cycle but serves to generate the active site Pi species. Orthophosphate remains in the active site for multiple rounds of catalysis until it eventually dissociates and ATP must rebind^19^. We furthermore demonstrated that ATP hydrolysis in the active site of Dop is dependent on the C-terminal residue of Pup, be it glutamate or glutamine^19^. We previously solved a crystal structure of Dop from *Acidothermus cellulolyticus* (^Acel^Dop) with ADP and Pi bound in the active site^19^ (PDB 5LRT), but a structure of Dop in complex with Pup has not been determined so far, and the mechanistic role played by the C-terminal residue of Pup in ATP hydrolysis is not known.

The unique features of the two homologous enzymes, PafA and Dop, that allow them to catalyze opposing reactions in the pupylation-depupylation cycle are still not fully understood. A recent study demonstrated that multiple mutational paths likely led to the emergence of the depupylase from the ligase activity, and that this path cannot easily be reenacted in vitro by reciprocal mutagenesis of uniquely conserved residues^20^.

One characteristic feature of the depupylase Dop that is absent in the homologous PafA ligase is a highly conserved sequence stretch of 40-65 residues preceding the β2 strand of the β-sheet cradle, which has been termed the Dop-loop^16^. Although it was shown that deletion of this region does not abolish Dop activity^16^, the high degree of sequence conservation in the first half of the loop would suggest that it plays a functional role. In the Dop structures solved so far, the Dop-loop could not be resolved, indicating that it was disordered^16,19^. However, it is possible that the Dop-loop undergoes a disorder-to-order transition during the catalytic cycle or under specific conditions. For example, the intrinsically disordered Pup undergoes such a disorder-to-order transition, forming two orthogonal helices in its last 27 residues upon binding to a long groove on PafA^17^. A homologous groove is also present in Dop. Differences in the shape of the groove and residues lining it as well as the fact that Pup binds to Dop an order of magnitude more tightly than to PafA^16^ indicate that Pup and Dop must exhibit some distinct interaction features.

In this study, we present a series of crystal structures of the depupylase Dop in complex with Pup in different intermediate states along the catalytic cycle. In combination with biochemical experiments, our analysis reveals the mechanism of ATP hydrolysis that is required to generate the catalytic phosphate in the active site, and provides insights into the allosteric regulatory role of the Dop-loop on Pup-binding and ATP hydrolysis.

## Results and discussion

### The Pup C-terminal residue forms extensive contacts in the Dop active site

**Fig. 1:**
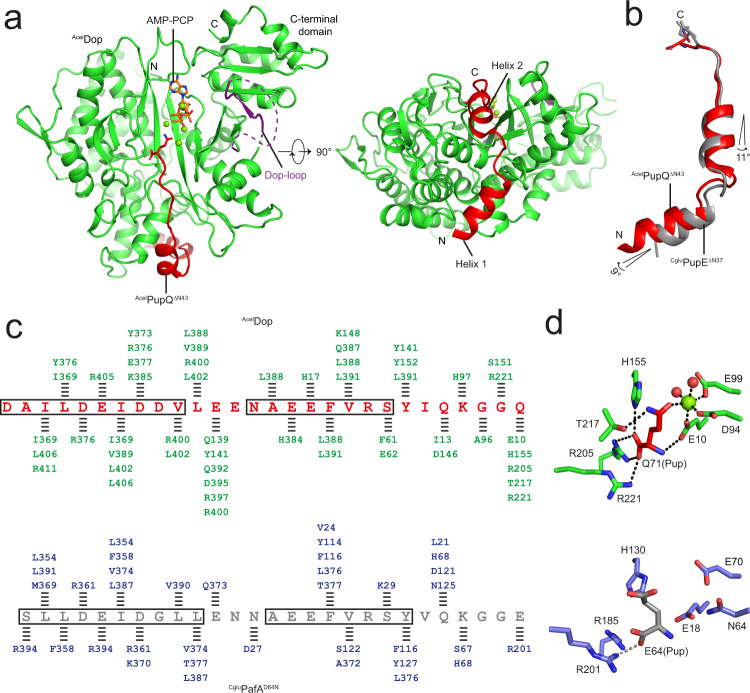
Pup bound to Dop exhibits a more extensive network of interactions than Pup bound to ligase PafA. **a** Overview of the Dop-PupQ-AMP-PCP complex structure. In the complex, Dop is colored green, PupQ^ΔN43^ red, the partially resolved Dop-loop purple, and AMP-PCP is colored orange. The disordered part of the Dop-loop is represented as a purple dashed line and magnesium ions are represented as green spheres. **b** Comparison of Pup structures when bound to Dop (red) or to PafA (gray, PDB 4BJR). Dop and PafA are omitted for clarity. The relative rotation angles were measured between the central axes of the helices. **c** Schematic comparison of the interactions formed by Pup in the Dop-Pup and in the PafA-Pup complex. Sequences of Pup are colored red (^Acel^ PupQ^ΔN43^) or gray (^Cglu^PupE^ΔN37^), and the residues forming the two orthogonal helices (helix 1 and helix 2) are outlined by black boxes. Dop-Pup and PafA-Pup interactions are indicated as black dashed lines, including hydrogen bonds, hydrophobic and electrostatic interactions assigned with a distance cut-off of 4 Å. Dop residues are colored green and PafA residues blue. **d** Molecular interactions formed by the C-terminal residue of Pup with Dop (green) or with PafA (blue). Only interacting residues are displayed and are shown in stick representation. Polar interactions are represented as black or gray dashed lines.

In order to deduce the catalytic mechanism of the depupylase enzyme, a structure of Dop in complex with Pup is crucial. This is particularly important, since the binding of Pup to Dop stimulates the generation of the inorganic phosphate that must be present in the active site for catalysis^19^. As previous experiments have shown that the N-terminal portion of Pup does not participate in binding to Dop^16^, an N-terminally truncated Pup-fragment, ^Acel^PupQ^ΔN43^, was used for co-crystallization experiments with ^Acel^Dop. Furthermore, in order to prevent the turnover of PupQ to PupE, the non-hydrolyzable ATP analog AMP-PCP was used, since ATPγS and AMP-PNP can still be hydrolyzed by Dop, albeit much more slowly. We obtained crystals of the enzyme in complex with the PupQ fragment and AMP-PCP and determined the structure at 1.65 Å resolution by molecular replacement (Fig. 1a; Table 1). The final atomic model contained all residues of PupQ^ΔN43^ bound to the enzyme (Supplementary Fig. 1).

**Table 1:** Data collection and refinement statistics.

	Dop-PupQ-AMP-PCP	Dop-PupE-ADP-MgF_3_(H_2_O)‾	Dop-PupE^P^-ADP	Dop-PupE-ADP-MgF_4_^2−^	Dop (Dop-loop-inserted)
PDB ID	7OXY	7OYF	7OY3	7OYH	7OXV
Crystal form					
Space group	*P 3*_*1*_ *2 1*	*P 3*_*1*_ *2 1*	*P 3*_*1*_ *2 1*	*P 3*_*1*_ *2 1*	*P 4*_*1*_*2*_*1*_*2*
Unit cell	106.668	106.334	106.806	106.827	72.533
dimensions (Å)	106.668	106.334	106.806	106.827	72.533
	108.791	107.384	108.457	107.947	184.149
angels α, β, γ (°)	90 90 120	90 90 120	90 90 120	90 90 120	90 90 90
Molecules/ASU	1	1	1	1	1
Data collection					
Wavelength (Å)	1.000	1.000	0.815	1.000	1.000
Resolution (Å)	46.19–1.65	47.65–1.88	46.78–1.78	47.87–1.75	46.04–1.39
	(1.709–1.65)	(1.947–1.88)	(1.844–1.78)	(1.813–1.75)	(1.444–1.39)
Total reflections	1,743,812 (177,795)	1,162,055 (111,367)	1,419,633 (139,117)	1,445,395 (146,848)	2,479,743 (216,822)
Unique reflections	86,165 (8,506)	57,433 (5,667)	68,753 (6,798)	72,028 (7,143)	98,553 (9,612)
Multiplicity	20.2 (20.9)	20.2 (19.7)	20.6 (20.5)	20.1 (20.6)	25.2 (22.6)
Completeness (%)	99.98 (99.99)	99.98 (99.98)	99.92 (99.96)	99.94 (99.89)	99.85 (98.99)
Mean I/σ(I)	21.30 (1.80)	17.14 (2.03)	12.56 (1.38)	17.52 (2.33)	26.76 (1.24)
Wilson B-factor (Å^2^)	27.75	29.56	24.81	23.38	19.73
R_merge_	0.084 (1.858)	0.124 (1.625)	0.168 (1.178)	0.150 (1.934)	0.069 (2.239)
R_meas_	0.086 (1.905)	0.128 (1.668)	0.172 (1.208)	0.154 (1.983)	0.070 (2.290)
CC_1/2_	1 (0.746)	0.999 (0.804)	0.999 (0.774)	0.999 (0.798)	1 (0.587)
Refinement					
Reflections used	86,152 (8,505)	57,429 (5,666)	68,738 (6,797)	71,997 (7,135)	98,538 (9,599)
Reflections used R_free_	4,381 (410)	2,960 (279)	3,271 (300)	3,621 (346)	4,965 (475)
R_work_	0.1729 (0.2629)	0.1592 (0.2178)	0.1701 (0.2808)	0.1619 (0.2391)	0.1334 (0.2561)
R_free_	0.1951 (0.2774)	0.1878 (0.2676)	0.1955 (0.2880)	0.1842 (0.2696)	0.1691 (0.3181)
Model composition					
Non-hydrogen atoms	4,490	4,462	4,595	4,638	4,805
Macromolecules	4,045	4,050	4,031	4,087	4,283
Ligands	119	111	101	177	68
Water	326	301	463	374	454
Protein residues	501	501	499	499	501
RMSD					
Bonds	0.007	0.010	0.007	0.007	0.009
Angles	0.89	1.10	0.90	0.92	1.09
Ramachandran plot					
Favored (%)	98.38	98.58	98.37	98.37	98.39
Allowed (%)	1.62	1.42	1.63	1.63	1.61
Outliers (%)	0.00	0.00	0.00	0.00	0.00
Rotamer outliers (%)	0.23	0.23	0.23	0.00	0.86
Clashscore	3.87	3.63	6.61	4.13	4.10
Average B-factor	33.43	33.94	30.59	28.46	28.33
Macromolecules	32.44	33.00	29.37	26.99	26.49
Ligands	47.52	50.35	41.78	43.81	67.64
Water	40.57	40.50	38.74	37.29	39.71

Values in parentheses are for highest-resolution shell.

${{{\mbox{R}}}}_{{{\mbox{merge}}}}=\sum \left|{I}_{h,i}-\left\langle {I}_{h}\right\rangle \right|/\sum {I}_{\left(h,i\right)}$, where $\left\langle {I}_{h}\right\rangle$ is the mean intensity of the reflections.

Upon binding to Dop, Pup undergoes a disorder-to-order transition adopting two well-resolved helices (helix 1: D44–V53; helix 2: N57–S64) (Fig. 1a, b). Comparison with the Pup-ligase complex (^Cglu^PafA^D64N^-^Cglu^PupE^ΔN37^-ATP, PDB 4BJR)^17^ shows that the overall conformation of Pup in the enzyme binding groove is conserved (Fig. 1b). However, Pup in complex with Dop exhibits a significantly more extensive network of interactions than Pup bound to the ligase PafA, with differences especially pronounced at the C-terminal residue of Pup (Fig. 1c, d). The C-terminal glutamine residue of Pup in the Dop-Pup complex is well defined in the electron density map (Supplementary Fig. 1) and forms extensive interactions with Dop (Fig. 1d, upper panel), whereas in the PafA-Pup complex, the C-terminus of Pup is poorly ordered, as indicated by the weak electron density^17^, and only weakly forms a salt bridge with the side chain of R201 (Fig. 1d, lower panel).

Depupylase Dop is structurally homologous to Pup ligase PafA, although the two evolutionarily related enzymes catalyze opposing reactions. It is possible that the tighter binding of the C-terminal Pup residue to the Dop active site compared to PafA contributes to the opposite directionality of the reaction catalyzed by Dop. A similar phenomenon was observed for the regulation of phosphoribosyl-linked (PR) serine ubiquitination by SidE (ubiquitinase) and DupA/B (deubiquitinase), which have catalytic domains that are highly homologous to each other yet catalyze chemically opposite reactions^21^. The deubiquitinase activity of DupA/B is favored by the high affinity of the PR-ubiquitinated substrate, whereas the ubiquitination activity of SidE is conferred by low-affinity interactions with ubiquitin. DupA can be converted into a SidE-type ubiquitin ligase by weakening the binding affinity of the ubiquitinated peptide. The Dop-Pup complex structure solved here shows that the loop preceding strand β7, referred to in the literature as α-loop (F208–K220)^20^, is involved in the binding of the C-terminal residue of Pup (Q71) through a strong hydrogen bond formed with T217 (Fig. 1d, upper panel). In comparison, the equivalent region (H188–S200) in the PafA-Pup complex structure (PDB 4BJR) forms an α-helix which does not interact with Pup^17^. Substitution of the α-loop with the equivalent region from PafA was reported to contribute to the conversion of the depupylase into a ligase^20^, thus supporting our hypothesis that the observed network of interactions constraining the C-terminal residue of Pup in the active site contributes importantly to the directionality of the catalyzed reaction.

### Allosteric interplay between Pup-binding and the Dop-loop

**Fig. 2:**
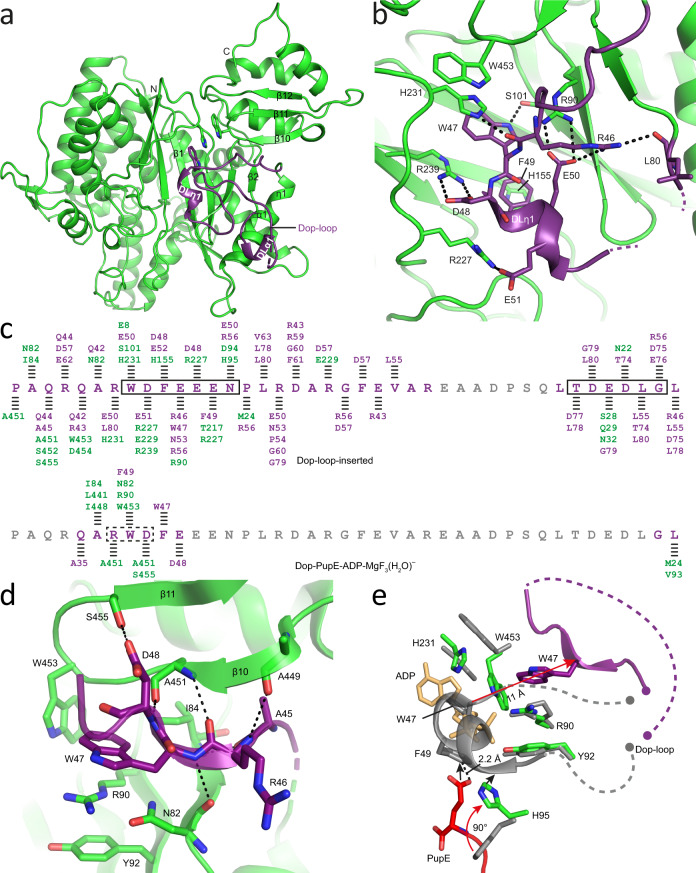
Interplay between Pup-binding and the Dop-loop conformation. **a** Crystal structure of Dop with the Dop-loop inserted into the empty active site. Dop is colored green except for the Dop-loop that is colored purple. The unresolved 7-residue stretch of the Dop-loop is depicted as a purple dashed line. **b** Close-up of the molecular interactions mediated by the first helix (DLη1) of the Dop-loop. Polar interactions are represented as black dashed lines. **c** Comparison of the interactions mediated by the Dop-loop in different conformations. Sequence of the Dop-loop in the resolved region is colored purple while for the disordered part it is colored gray. The regions forming helices or β-strands were outlined as solid or dashed black boxes, respectively. Interactions between Dop (green) and the Dop-loop (purple) are indicated as black dashed lines, including hydrogen bonds, hydrophobic and electrostatic interactions assigned with a distance cut-off of 4 Å. **d** Zoomed-in view of the interactions between the resolved Dop-loop region (purple) and Dop (green) as observed in all Pup-bound structures in this study (here shown on the example of the Dop-PupE-ADP-MgF_3_(H_2_O)^−^ structure). Polar interactions are represented as black dashed lines. **e** Structural comparison of active site residues as observed in the Dop-loop-inserted structure (gray) and a Pup-bound Dop structure (shown on the example of the Dop-PupE-ADP-MgF_3_(H_2_O)^−^ complex) (green, red, light-orange and purple). Red arrows indicate the movements induced by Pup-binding, while black arrows suggest strong steric clashes. The unresolved or omitted parts of the Dop-loop were depicted as dashed lines (gray or purple) with the ends of the loop represented as filled dots.

The sequence stretch preceding strand β2 and referred to as the Dop-loop is a distinguishing feature between the depupylase and the Pup ligase, since it is present in all Dop orthologs but absent in all PafA homologs^16^. In previously published Dop structures, the Dop loop was not resolved, however, given its high degree of conservation (Supplementary Fig. 2a), it is likely that the Dop-loop adopts a defined conformation at specific intermediate states during catalysis. With the aim to capture Dop in a state where the Dop-loop could be resolved, crystallization experiments were carried out with ^Acel^Dop, exploring conditions with Dop alone and Dop in complex with Pup and/or different adenine nucleotides. We were successful in identifying crystallization conditions, where ^Acel^Dop in the absence of other ligands features the Dop-loop in a well-resolved conformation that is not induced via crystal contacts (Fig. 2a; Supplementary Figs. 2b and 3a; Table 1).

In this structure, 33 of the 40 residues in the Dop-loop are resolved (Supplementary Fig. 2b), including residues P40–R65 and L73**–**L80 and thereby all of the highly conserved residues in the loop (Supplementary Fig. 2a). The Dop-loop features two very short helical stretches (DLη1 and DLα1) flanking a long zigzagging loop (Fig. 2a). Helix DLη1 (η stands for 3_10_ helix) consists of residues 48-52 (DFEEE) and is located nearby the nucleotide-binding pocket (NBP) of Dop, while DLα1 is formed by residues 75-78 (DEDL) and is positioned distal to the active site. The Dop-loop forms extensive interactions with residues surrounding the active site (Fig. 2b, c, upper panel). In particular, insertion of the highly conserved Dop-loop-residue W47 into the NBP leads to the formation of a triple π-stack, where W47 interacts with H231 that in turn stacks with W453 of the C-terminal domain (Fig. 2b). Another π-stacking interaction is formed by Dop-loop-residue F49 and H155. Furthermore, a network of salt bridges is formed between acidic residues in the Dop-loop and several arginine side chains surrounding the active site.

In contrast, in the Pup-bound structure described in the previous section and in the Pup-bound structures described in later sections of this article, which we obtained in crystallization efforts with metal fluorides, the Dop-loop is mostly disordered except for the highly conserved residue W47 and six residues flanking it (Supplementary Fig. 3b). Interestingly, in the Pup-bound structures, W47 stacks with the side chain of R90 and forms a hydrophobic interaction with the conserved W453 residue of the C-terminal domain (Fig. 2c, lower panel, and 2d; Supplementary Fig. 2a), indicating that Pup-binding significantly reorders the Dop-loop. Superimposition of the Dop-loop-inserted structure with any of the Pup-bound structures reveals dramatic conformational changes of the Dop-loop and several active site residues. Although the conformation of the visible stretch of the Dop-loop is congruent in all the Pup-bound structures we solved, the Pup-bound complex with ADP-MgF_3_(H_2_O)^−^ in the active site, which is discussed more extensively in a later paragraph of this article, is used here for superimposition due to the fact that it exhibits the highest quality map for the partially resolved Dop-loop (Fig. 2e; Supplementary Fig. 3b). The comparison shows that, upon binding of Pup, H95 swings upward by about 90° out of the way of Pup’s C-terminal residue and displaces the Dop-loop, including residue W47 that moves around 11 Å away from the NBP. To complement our crystallographic study with biochemical analysis, we generated a Dop-loop variant of Dop from *Corynebacterium glutamicum* (^Cglu^Dop), referred to as ^Cglu^DopGS, where the Dop-loop (H43–I77) was replaced with a short linker sequence (GS)_4_. ^Cglu^Dop was chosen for the in vitro biochemical and mutational analysis due to its higher solubility compared to the mycobacterial orthologs and because *C. glutamicum*, in contrast to *A. cellulolyticus*, is not a thermophile. Using isothermal titration calorimetry (ITC) binding experiments, we show that PupE binds to the DopGS variant with an affinity ~2.5 times tighter than to the wild type (WT) enzyme (Supplementary Fig. 3c and 3d), supporting the notion that Pup binding is coupled to the displacement of the Dop-loop.

### The Dop-loop affects ATP hydrolysis allosterically

When Pup is bound to the active site, the highly conserved Dop-loop residue W47 stacks with the side chain of R90 via a cation-π interaction (Supplementary Fig. 4a). R90 in turn stacks with the side chain of Y92, stabilizing the binding of nucleotide in the active site of Dop through a salt bridge with the α-phosphate group. In this conformation W47 influences the positioning of nucleotide in the active site and could thereby affect the ATP hydrolysis that generates the catalytic phosphate and must take place before the catalytic cycle of Dop can begin.

**Fig. 3:**
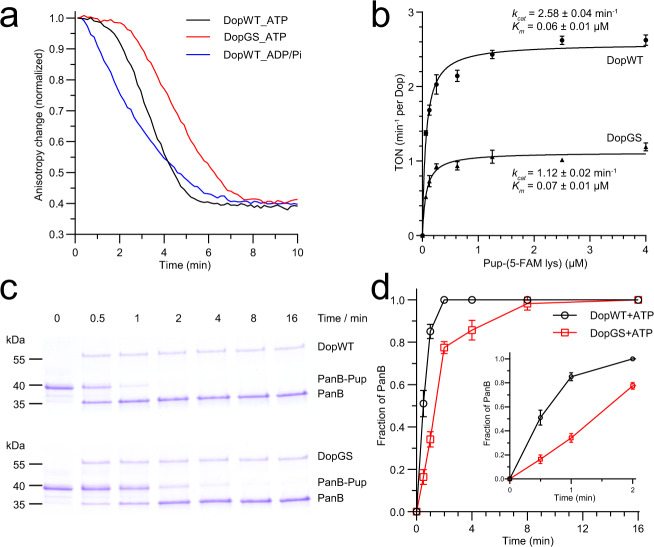
ATP hydrolysis of Dop is allosterically influenced by the Dop-loop. **a** The Dop-loop variant (DopGS) exhibits a longer phase in the depupylation time course of the fluorescent model substrate Pup-(5-FAM-Lys) (Pup-Fl) than the wild type (WT) enzyme in the presence of ATP. 1.25 µM ^Cglu^Dop (WT or DopGS variant) was incubated with 5 μM Pup-Fl and 100 µM ATP or ADP plus 10 mM Na/K Pi (pH 8.0 at 23 °C) at 30 °C. The measurement was started by addition of nucleotide. Each line represents the average of three independent replicates. **b** Steady-state enzyme kinetic analysis of depupylation of Pup-Fl by using fluorescence anisotropy. Each experiment was carried out in three independent replicates and data are represented as mean values ± SD. **c** Depupylation time courses of PanB-Pup catalyzed by Dop variants in the presence of ATP. PanB-Pup (3 µM) was incubated with 0.5 µM ^Cglu^Dop (DopWT or DopGS) at 30 °C supplemented with 0.5 mM ATP. **d** Densitometric analysis of PanB bands in (**c**) with respect to the total amount of PanB-Pup used in the reaction. Each reaction was carried out in two or three independent replicates and data are represented as mean values ± SD.

Using the fluorescent model substrate Pup-(5-FAM-Lys) (Pup-Fl) it is possible to follow the isopeptidase activity of Dop by measuring the resulting decrease in fluorescence anisotropy^22^. In the presence of ATP but not ADP/Pi, ^Cglu^DopWT exhibits a lag phase that was previously shown to correlate with the production of ADP and Pi in the active site^19^ (Fig. 3a). The DopGS variant exhibits a prolonged lag phase, suggesting an allosteric influence of the Dop-loop on ATP hydrolysis. Pup-Fl turnover catalyzed by the DopGS variant is slower than the turnover catalyzed by WT Dop, as is evident from the steeper slope in the anisotropy time course of the WT enzyme. In order to determine the effect of a missing Dop-loop on the steady-state parameters of isopeptide bond cleavage by Dop, we carried out turnover measurements as a function of Pup-Fl concentration and analyzed them according to Michaelis-Menten kinetics (Fig. 3b). While the *K*_*m*_ remains unchanged, the *k*_*cat*_ of depupylation in the presence of ATP exhibited by the DopGS variant is 2.3-fold lower than that of the WT enzyme. We also compared WT Dop and the DopGS variant by following depupylation of pupylated ketopantoate hydroxymethyltransferase (PanB-Pup) using a gel-based assay (Fig. 3c, d). Although this assay does not offer the same resolution as the anisotropy assay, our data show that the DopGS variant is lagging behind the WT enzyme in the PanB-Pup depupylation time course. Taken together, our results indicate that the Dop-loop aids formation of the active site Pi species and also enhances the overall turnover of pupylated substrate.

A recent study suggested that the Dop‐loop serves as a regulatory element that inhibits the depupylation activity^23^, however, such an inhibitory role of the Dop-loop on depupylation was not observed in our experiments (Fig. 3). Our results are more in line with the observation that the removal of the Dop-loop in addition to the substitution of the α-loop from PafA can convert the depupylase Dop into a ligase^20^, as our results indicate that the Dop-loop stimulates the production of the active site phosphate, an important step for enabling C–N bond cleavage.

### ATP γ-phosphate and the Pup C-terminal residue are poised for nucleophilic attack

Although we previously demonstrated that ATP hydrolysis in the active site of Dop is dependent on Pup binding^19^, the mechanistic role played by Pup in ATP hydrolysis remained unknown. Structural analysis of Pup-bound Dop complexes should provide the molecular basis for understanding the role of Pup in the production of the catalytic phosphate.

In the active site of the Dop-PupQ complex (Supplementary Fig. 4a), the non-hydrolyzable ATP analog AMP-PCP displays strong and unambiguous density with an occupancy of about 72 % (Supplementary Fig. 4b). Correspondingly, two of the three canonical Mg^2+^-binding sites (n1–n3) defined for the carboxylate-amine/ammonia ligase superfamily also show only partial occupancies (n2 and n3).

**Fig. 4:**
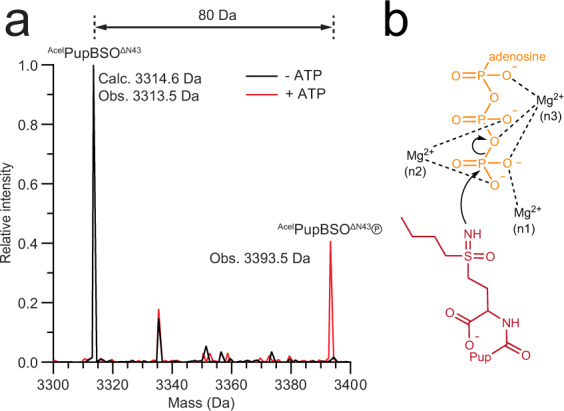
Dop phosphorylates a Pup derivative carrying a C-terminal buthionine sulfoximine (BSO). **a** Mass spectrometry analysis reveals the production of phosphorylated PupBSO by Dop in the presence of ATP. **b** Proposed reaction mechanism of ^Acel^PupBSO^ΔN43^ phosphorylation by Dop in the presence of ATP.

The γ-phosphate of AMP-PCP is kinked slightly out of the active site and away from the side chain of the C-terminal Pup residue (Supplementary Fig. 4a), likely due to the inability of the AMP-PCP-analogue-specific carbon atom between the β- and γ-phosphates to fill the empty coordination position of Mg^2+^ at the n3 site. In the structure of GS solved in the presence of AMP-PNP (PDB 2D3B)^24^, the equivalent γ-phosphate is oriented towards the side chain of the bound inhibitor methionine sulfoximine (MSO) and the bridging nitrogen atom coordinates the manganese (Mn^2+^) ion at the n3 site (Supplementary Fig. 4c). In the presence of ATP, the NH group of MSO makes a nucleophilic attack on the γ-phosphorus of ATP producing phosphorylated MSO^24^. As Dop features a highly similar active site configuration as GS (compare Supplementary Fig. 4a and 4c), it is expected that AMP-PNP binds to Dop in the same configuration as to GS. In order to determine experimentally if the γ-phosphate group adopts a similar conformation in Dop, we tested if Dop is also able to react with a sulfoximine inhibitor. We used a Pup derivative (^Acel^PupBSO^ΔN43^) whose C-terminal residue was replaced with buthionine sulfoximine (BSO), a potent inhibitor of glutamate cysteine ligase (GCL)^25,26^. Using intact mass spectrometry, we show that Dop indeed forms a phosphorylated PupBSO species in the presence of ATP (Fig. 4), demonstrating that the γ-phosphate is oriented towards the C-terminal residue of Pup and stabilized by coordination with Mg^2+^ at the canonical n1–n3 sites.

### Production of Pi is preceded by phosphoryl transfer to C-terminal Pup residue

**Fig. 5:**
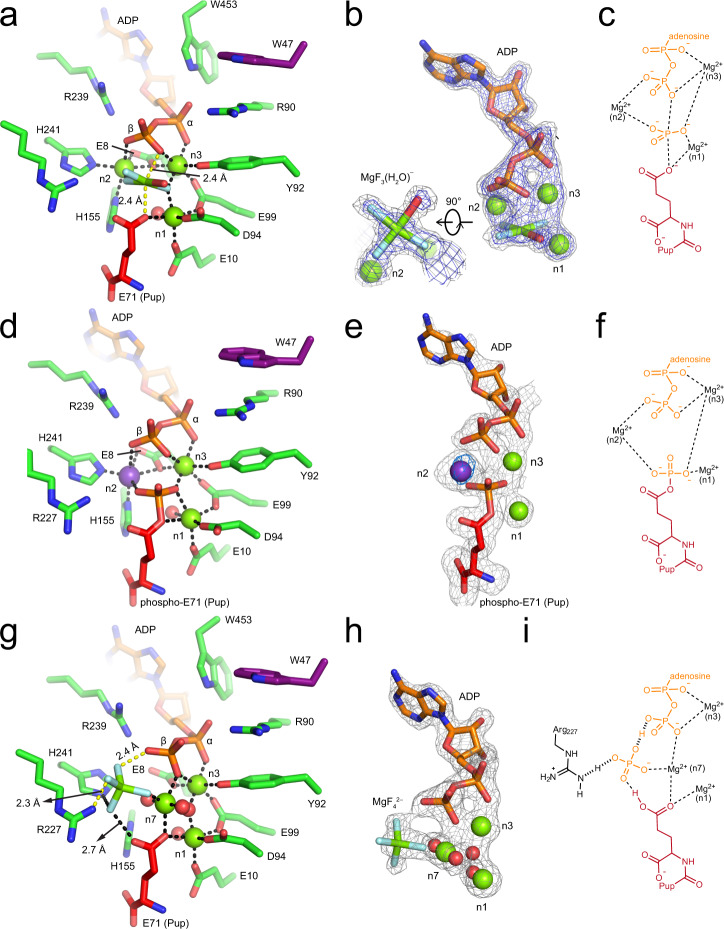
Crystal structures of Dop-PupE complexes in distinct intermediate states of ATP hydrolysis generating the catalytic Pi in the active site. **a**-**c**, Crystal structure of the Dop-PupE-ADP-MgF_3_(H_2_O)^−^ complex mimics the transition state of ATP hydrolysis. **a** Dop active site with bound ADP (orange), magnesium ions (green spheres), the C-terminal residue of Pup (red) and MgF_3_(H_2_O)^−^ (green, cyan, and red). The color-coding of the Dop structure here is consistent with Fig. 1a. Polar interactions are indicated by black or yellow dashed lines. **b** The unbiased mFo-DFc Fourier map of ADP, magnesium ions, and MgF_3_(H_2_O)^−^ at 4.0 σ (gray) or 5.7 σ (blue) contour level was calculated during molecular replacement using PDB 5LRT excluding ligands followed by three rounds of refinement without further model building. **c** Schematic representation of the proposed transition state of ATP hydrolysis in the Dop active site. **d**–**f** Crystal structure of Dop in complex with phosphorylated PupE^ΔN43^. **d** Active site of Dop with bound ADP, phosphorylated PupE^ΔN43^, magnesium and potassium ions (purple sphere). Interactions are presented as black dashed lines. **e** The simulated annealing polder map of ADP, magnesium ions, potassium ion and Pup C-terminal residue at 3.0 σ contour level was calculated using *phenix.refine* with the coordinates of the input model randomly displaced by 0.5 Å. The blue density at 5 σ contour level around the potassium ion represents the anomalous signal calculated from a data set collected at 2 Å wavelength. **f** Proposed active site of Dop in complex with ADP, magnesium ions and phosphorylated Pup. **g**–**i** Crystal structure of Dop-PupE-ADP-MgF_4_^2−^ complex mimics the product state of ATP hydrolysis. **g** Dop active site with bound ADP, magnesium ions, MgF_4_^2−^ (green and cyan) and the C-terminal residue of Pup. Polar interactions are represented as black dashed lines except the low-barrier-hydrogen-bonds (yellow dashed lines). **h** The unbiased mFo-DFc Fourier map of ADP, magnesium ions, MgF_4_^2−^ and water molecules at 3.0 σ contour level was calculated the same way as for Fig. 5b. **i** Schematic representation of the proposed product state of ATP hydrolysis in the active site of Dop. Shared protons are bonded as hashed lines.

The mechanism of enzyme-catalyzed ATP hydrolysis has been widely studied by using metal fluorides to substitute the phosphoryl group in order to stabilize distinct catalytic intermediates^27^. While the tetrahedral beryllium trifluoride (BeF_3_^−^) mimics the ground state, the octahedral aluminum tetrafluoride (AlF_4_^−^) and trigonal bipyramidal magnesium trifluoride (MgF_3_^−^) present transition state analogs^28,29^. In order to gain structural insights into ATP hydrolysis generating the active site inorganic phosphate species^19^, we carried out extensive screens to co-crystallize the Dop-Pup complex with different metal fluorides. Magnesium fluoride (MgF_*x*_) proved to be best suited, yielding soluble complexes and producing well-diffracting crystals. We solved the structure of a Dop-Pup complex with a square planar MgF_*x*_ species bound between ADP and Pup (Fig. 5a, b; Table 1). The same unexpected square planar configuration was recently observed in the structure of Zika virus NS3 helicase crystallized in the presence of MgF_*x*_^30^. It was identified as containing three fluorines and one water molecule, MgF_3_(H_2_O)^−^, in the equatorial plane. In Dop, detailed examination of the unbiased omit map of the compound revealed weaker electron density at the site closest to D94, thereby identifying it as oxygen (Fig. 5a, b).

The MgF_3_(H_2_O)‾ species mimicking the dissociating γ-phosphate in the Dop active site is sandwiched at equal distance of around 2.4 Å from the β-phosphate of ADP and from the carboxylate side chain of the C-terminal Pup residue, forming an octahedral coordination geometry (Fig. 5a). Two of the fluorine atoms are coordinated to Mg^2+^ ions in the active site, one to the n2 site Mg^2+^ and the other held between the n1 and n2 site Mg^2+^ ions, which helps to neutralize the charges developing on the γ-phosphate of ATP during hydrolysis. The configuration of MgF_3_(H_2_O)^−^ is suggestive of a transition state for phosphoryl transfer to the C-terminal glutamate residue of Pup^30^ (Fig. 5a, c). Indeed, our structure agrees with a transition state model proposed previously for GS-catalyzed glutamine synthesis^24^. The PupE fragment used here for co-crystallization with Dop represents the product generated after deamidation or depupylation. We previously showed that both PupQ and PupE can stimulate the formation of the active site inorganic phosphate species from ATP^19^. It is therefore expected that the analogous transition state complex is formed with PupQ and magnesium fluoride. Taken together, our structure suggests that the C-terminal amide or carboxylate side chain of Pup is directly involved in ATP cleavage and presents the acceptor group in the transition state for phosphoryl transfer.

The phosphorylated Pup intermediate resulting from phosphoryl transfer to the amide side-chain of the C-terminal residue should only exist transiently in solution and be readily hydrolyzed after its formation^19^. However, when crystals of Dop-PupE complex were soaked with ATP for 19 h, we serendipitously produced Dop with a bound phosphorylated Pup fragment and ADP in the active site (Fig. 5d, e; Table 1). Presumably due to the high concentration of KH_2_PO_4_ in the crystallization solution, a potassium (K^+^) ion instead of a Mg^2+^ ion was identified at the n2 site, as indicated by the strong anomalous signal peak from data collected at around 2.0 Å wavelength (Fig. 5e), although under physiological conditions the n2 site is most likely occupied by a Mg^2+^ ion (Fig. 5f). However, the binding of K^+^ at the n2 site does not affect the binding of ADP and the C-terminal Pup residue, as both bind in a similar conformation as observed in the transition state complex (Supplementary Fig. 5). Furthermore, the active site with the phosphorylated Pup intermediate displays a similar configuration as the active site of GS complexed with ADP, Mn^2+^ and phosphorylated MSO (PDB 2D3A)^24^. This further supports a mechanism involving the nucleophilic attack of the side chain carboxylate oxygen of the C-terminal Pup residue on the γ-phosphate of ATP proceeding through a transition state of phosphoryl transfer^24^ as observed in the Dop-Pup structure in complex with ADP and octahedral trifluoromagnesate (Fig. 5a).

### Active site Pi is held in place for multiple rounds of catalysis by two low-barrier hydrogen bonds

In order to observe the Dop active site after generation of the catalytic phosphate from the phospho-Pup intermediate, we attempted to obtain crystals of Dop-Pup-ADP in complex with sulfate or tetrafluoromagnesate, both of which could theoretically serve as Pi mimics^31,32^. Dop-Pup-ADP crystals were soaked with buffers containing high concentrations of MgSO_4_ and NaF, allowing us to determine a structure of the product state with a clear tetrahedral electron density observed for the Pi analog (Fig. 5g, h; Table 1). Structure refinement with SO_4_^2−^ or MgF_4_^2−^ placed in the tetrahedral electron density (Supplementary Fig. 6a and 6b) showed that although both of the ligands were refined to partial occupancies (65 % vs 74 %, respectively), MgF_4_^2−^ fits the observed density better than SO_4_^2−^ and was hence modeled in the final structure of the product state (Fig. 5g, h).

The binding of the tetrahedral MgF_4_^2−^ in the active site of Dop is mediated via an intricate polar interaction network. The contacts include electrostatic interactions formed with the side chain of the highly conserved R227 and the Mg^2+^ ion bound at the site where the square planar magnesium fluoride is located in the transition state complex (referred to here as n7 site) (Supplementary Fig. 6c), as well as hydrogen bonds established with the side chain of H241, the C-terminal residue of Pup and likely the β-phosphoryl oxygen of ADP (Fig. 5g). Notably, the distance between MgF_4_^2−^ and the β-phosphate of ADP is only 2.4 Å. Although we cannot confirm the existence of a hydrogen atom in a density map at 1.75 Å resolution, it stands to reason that in the absence of a hydrogen bond, such a short distance would lead to strong electrostatic repulsion. We, therefore, conclude that MgF_4_^2−^ interacts with the β-phosphate of ADP through a hydrogen bond. Hydrogen bonds with a length below 2.6 Å are particularly strong, and this type of hydrogen bond, referred to as low-barrier hydrogen bond (LBHB), is frequently found in the active sites of enzymes^33–35^. Another LBHB identified in this structure is located between MgF_4_^2−^, the Pi mimic, and R227, separated by a distance of around 2.3 Å (Fig. 5g). Therefore, it appears that Pi in the active site of the Dop-Pup complex is held by two strong LBHBs (Fig. 5g, i), likely preventing fast dissociation of Pi, which explains the observation that the generated ADP/Pi in the active site can support multiple rounds of depupylation/deamidation^19^.

Our earlier hypothesis for the catalytic role of Pi during depupylation was that Pi acts as a nucleophile attacking the isopeptide bond of a pupylated substrate to form a phosphorylated Pup intermediate^19^. Based on the structure presented here of the Dop-PupE complex with the Pi mimic in the active site, Pi could alternatively serve to make the carbonyl carbon of the amide a better electrophile by stabilizing the amide resonance form with the carbon-nitrogen double bond. This could allow the direct nucleophilic attack of a water molecule to cleave the C–N bond. Supporting this alternative model is the location of MgF_4_^2−^, where an ideal Bürgi-Dunitz angle of attack (α_BD_)^36^ towards the side chain carbonyl group of the C-terminal Pup residue is not achieved, so that Pi might not be able to initiate a nucleophilic attack on Pup. However, we cannot rule out that Pi attacks the isopeptide bond, as we cannot be sure of the exact positioning of Pi in the Dop-PupQ complex (as opposed to the Dop-PupE complex we have solved here). Both scenarios are possible and in line with the mechanistic features of the γ-carboxylate/amine ligase superfamily, and in both cases, catalysis depends on the presence of the active site phosphate species.

### Mechanism of Pup-stimulated ATP cleavage to generate the catalytic Pi species

**Fig. 6:**
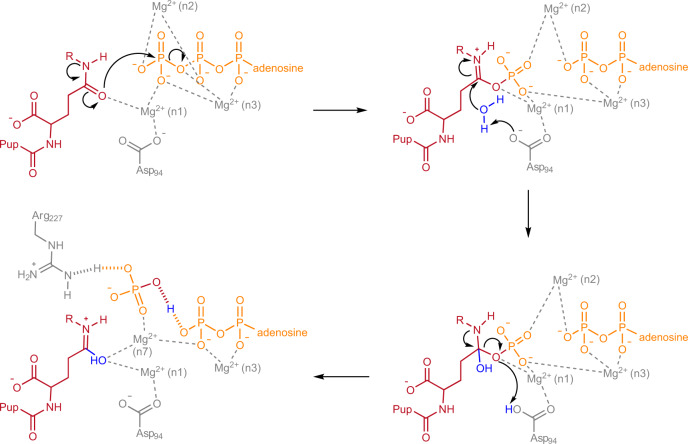
Proposed mechanism for the generation of the catalytic Pi in the active site of Dop. Active site residues are numbered according to ^Acel^Dop and shown in simplified representation omitting magnesium coordinating residues (Glu8, Glu10, Tyr92, Glu99, His155, and His241) and waters. Shared protons are bonded as hashed lines. R is equal to H or substrate.

Before the catalytic cycle of Dop can begin, Dop-mediated cleavage of ATP in the active site has to take place to generate the Pi species important for catalysis^19^. With the crystal structures of Dop-Pup complexes in all intermediate states of ATP hydrolysis in hand, including the ground state (Dop-PupQ-AMP-PCP) (Supplementary Fig. 4a and 4b), the transition state of phosphoryl transfer (Dop-PupE-ADP-MgF_3_(H_2_O)^−^) (Fig. 5a, c), the Pup-phosphorylated state (Dop-Pup^P^-ADP) (Fig. 5d, f) and the product state (Dop-PupE-ADP-MgF_4_^2−^) (Fig. 5g, i), we can develop a full mechanistic model for the generation of the catalytic Pi species (Fig. 6). We previously showed that the stimulation of Dop-mediated ATP hydrolysis by the C-terminal residue of Pup can be supported by both glutamate (E) and glutamine (Q) as C-terminal residue^19^. We, therefore, propose that both PupQ and PupE employ their C-terminal side-chain carbonyl oxygen to serve as the nucleophile attacking the γ-phosphorus of ATP (Fig. 6a). In case of PupE, this is the analogous reaction mechanism as occurs in GS during glutamine synthesis^24^. With the deamidation substrate PupQ (or the substrate-linked Pup) the C-terminal side chain amide would carry out this reaction in a similar fashion as is observed for argininosuccinate synthesis, where the side chain carbamide oxygen of citrulline nucleophilically attacks the α-phosphorus of ATP to form the activated acyl-AMP intermediate^37^. The C-terminal side chain amide of PupQ in the active site of Dop might exist as a significant resonance form of the carbon-nitrogen double bond, which is stabilized via coordination with the Mg^2+^ bound at the n1 site, thus making the amide carbonyl oxygen a good nucleophile for the γ-phosphorus of ATP. The carbamidyl phosphate moiety produced in the active site of Dop through a transition state of phosphoryl transfer is stabilized through coordination with three Mg^2+^ ions bound at the n1, n2 and n3 sites (Figs. 5c, f and 6). In the subsequent step, a nearby water molecule, most likely activated by the side chain of D94, is responsible for hydrolysis of the glutaminyl-phosphate intermediate^19^. The resulting Pi product is stabilized in the active site through strong LBHBs and through coordination with a Mg^2+^ bound at the n7 site (Figs. 5g, i and 6). In order to test our hypothesis, we mutated the C-terminal residue of Pup to methionine (PupM), which is of similar length as glutamine, but does not possess the amide functional group. ATP hydrolysis was measured in the absence of Pup, in the presence of PupE, and in the presence of PupM (Supplementary Fig. 7). PupM is unable to stimulate ATP hydrolysis in the Dop active site, demonstrating that the carboxylate/amide side chain of the C-terminal Pup residue is directly involved in ATP hydrolysis.

Taken together, our high-resolution X-ray crystallographic data supported by biochemical analysis provide comprehensive insights into the catalytic mechanism of Dop-mediated ATP hydrolysis, which proceeds via a transition state of phosphoryl transfer from the γ-phosphate of ATP to the C-terminal side chain carbonyl oxygen of Pup. Furthermore, we demonstrate that a characteristic loop in Dop (the Dop-loop) plays a key role in the process by serving as an allosteric sensor to detect Pup binding and subsequently promote ATP hydrolysis and catalysis.

## Methods

### Protein expression and purification

^Acel^Dop was expressed from isopropyl-β-D-1-thiogalactopyranoside (IPTG)-inducible vector pET21 in *Escherichia coli* Rosetta (DE3) cells (Invitrogen) as a C-terminal tobacco etch virus (TEV) protease cleavage site-His_6_ fusion for 16 h at 20 °C. For purification, cleared lysate was loaded on a 5 mL Hi-Trap immobilized metal affinity chromatography (IMAC) HP column (GE Healthcare Life Sciences) charged with Ni^2+^. The column was washed with 50 mL of buffer W (50 mM Tris-HCl pH 8.0 at 23 °C, 300 mM NaCl, 10 % (v/v) glycerol and 40 mM imidazole) and the protein was eluted with buffer W containing 300 mM imidazole. Dop-containing fractions were pooled and dialyzed overnight at 4 °C against buffer D (50 mM Tris-HCl pH 8.0 at 4 °C, 150 mM NaCl, 10 % (v/v) glycerol and 1 mM EDTA). The C-terminal His_6_-tag was cleaved at the TEV protease cleavage site by the addition of His-tagged TEV protease. TEV protease was subsequently removed via Ni^2+^-affinity chromatography. ^Acel^Dop was further purified by size exclusion chromatography using a Superdex 75 gel filtration column in 20 mM HEPES-NaOH pH 8.0 at 4 °C and 50 mM NaCl. ^Cglu^Dop and its variants with an N-terminal His_6_-TEV protease cleavage site fusion were expressed from pET24 and purified similarly to ^Acel^Dop except that the buffer used for size exclusion chromatography was changed to buffer D containing 300 mM NaCl, 10 % (v/v) glycerol, and 1 mM DTT. For biochemical assays, ^Cglu^Dop and its variants were further purified by anion exchange chromatography on a Mono Q 5/50 GL column (Cytiva) to reduce the amount of co-purified *E. coli* adenylate kinase (Adk). ^Cglu^Dop was eluted with a linear gradient from 0 to 1 M NaCl in 20 mM Tris-HCl pH 7.0 at 4 °C and exchanged into buffer D by dialysis.

PanB-Strep from *Mycobacterium tuberculosis* (Mtb) was expressed and purified as described previously^9,38^. ^Mtb^PanB-^Cglu^Pup was generated by in vitro pupylation as described before^10,38^. In short, PanB-Strep was expressed from an IPTG-inducible pETDuet plasmid in *Escherichia coli* Rosetta (DE3) cells (Invitrogen) for 16 h at 20 °C. After clearing the lysate, PanB-Strep was purified by Strep-Tactin XT Superflow column (IBA Lifesciences) followed by size exclusion chromatography on a Superose 6 pg column equilibrated and run in 50 mM Tris-HCl, pH 7.5 (4 °C), 150 mM NaCl, 1 mM DTT, 1 mM EDTA and 10 % glycerol. Pupylation reactions were performed in reaction buffer containing 50 mM HEPES-NaOH pH 8.0 at 23 °C, 150 mM NaCl, 10 % glycerol, 20 mM MgCl_2_, and 1 mM DTT supplemented with 5 mM ATP. 35 μM PanB-Strep, 50 μM PupE were incubated with 1 μM His_6_-TEV-^Cglu^PafA in several 500 μl reaction volumes at 30 °C for 18 h. PafA afterwards was removed by Ni^2+^-affinity chromatography and ^Mtb^PanB-^Cglu^Pup was stored in 50 mM HEPES-NaOH pH 8.0 at 23 °C, 300 mM NaCl and 10 % glycerol.

The Pup fragments (^Acel^PupE/Q^ΔN43^) used for crystallization were synthesized by GenScript, while ^Cglu^PupE or ^Cglu^PupM was expressed and purified as described previously^9^ with the addition of an anion exchange step to remove co-purified *E. coli* Adk. Briefly, Pup was expressed as a His_6_-Thioredoxin-TEV-Pup fusion protein and purified by a 5 ml Hi-Trap IMAC HP column (GE Healthcare Life Sciences) charged with Ni^2+^. After cleavage of the fusion protein with His-tagged TEV protease, His_6_-Thioredoxin and TEV protease were removed by Ni^2+^-affinity chromatography. Pup was further purified by size-exclusion chromatography on a Superdex 200 column (GE Healthcare) and an anion-exchange column (Mono Q 5/50 GL column, Cytiva), and stored in 50 mM HEPES-NaOH pH 8.0 at 23 °C, 150 mM NaCl, 1 mM EDTA and 0.5 mM TCEP. ^Acel^PupBSO^ΔN43^ peptide was synthesized by PEPSCAN.

### Gel-based depupylation assays

3 µM ^Mtb^PanB-^Cglu^Pup was incubated with 0.5 µM ^Cglu^Dop or ^Cglu^DopGS variant at 30 °C in buffer R (50 mM HEPES-NaOH pH 8.0 at 23 °C, 150 mM KCl, 10 % (v/v) glycerol, 1 mM DTT and 20 mM MgCl_2_) supplemented with 0.5 mM nucleotide and additional 10 mM Na/K phosphate (pH 8.0 at 23 °C) in the case of ADP. The formation of PanB was monitored by SDS-PAGE followed by Coomassie staining and was analyzed densitometrically using the GelAnalyzer software (version 2010a). The fraction of PanB is expressed with respect to the total PanB (sum of PanB and PanB-Pup). The contribution from Pup is negligible due to its small size and poor staining.

### Isothermal titration calorimetry

Isothermal titration calorimetry (ITC) measurements were performed at 25 °C on a MicroCal iTC200 instrument (GE Healthcare). ^Cglu^Dop (WT and DopGS variant) and ^Cglu^PupE were exchanged to buffer containing 50 mM Tris-HCl pH 8.0 at 4 °C, 300 mM NaCl and 20 mM MgCl_2_ by gel filtration. Titrations consisted of 22 injections of 1.75 µL ^Cglu^Dop (150 µM) or ^Cglu^DopGS (200 µM) to a cell containing 200 µL ^Cglu^PupE at a concentration of 15 or 20 µM, respectively. The acquired calorimetric titration curves were analyzed using Origin 7.0 (GE Healthcare) applying a 1:1 binding model.

### Electrospray ionization mass spectrometry of phosphorylated PupBSO species

To form the phosphorylated Pup species, ^Acel^PupBSO^ΔN43^ (4 μM) was mixed with ^Acel^Dop (3.4 μM) in the presence or in the absence of ATP (2 mM) in 50 mM Tris-HCl pH 8.0 (23 °C), 150 mM NaCl, 20 mM MgCl_2_ and 10 % glycerol. After 20 min of incubation at 30 °C, protein samples (50 μl) were desalted with Zip-Tip C4 pipette tips (Millipore). Desalted samples were eluted from C4-coated pipette tips with 50 μl of 50 % acetonitrile in water and loaded into the Synapt G2-Si mass spectrometer. The Sampling Cone energy was set at 40 V, and the capillary voltage was set at 3.0 kV. The neutral-mass spectra were inferred from the mass over charge measurement deconvolution using the MaxEnt3 Software.

### ATP hydrolysis assays

30 µM ^Cglu^DopWT was incubated with 1 mM ATP at 30 °C in 50 mM HEPES-NaOH pH 8.0 (23 °C), 150 mM KCl, 10 % (v/v) glycerol, 1 mM DTT and 20 mM MgCl_2_ for 5 min, before the hydrolysis reaction was started by addition of 40 µM ^Cglu^PupE or ^Cglu^PupM. Aliquots were drawn from the reaction after 15 min, mixed with urea to a final concentration of 6 M to stop the reaction and then analyzed by anion exchange chromatography (Resource Q, GE Healthcare) using a FPLC instrument (GE Healthcare). Nucleotide elution was followed by measuring absorbance at 260 nm and the ADP peak was integrated. The percentage of ATP hydrolyzed was calculated from the ADP generated during the reaction over the total amount of nucleotides.

### Fluorescence anisotropy-based steady-state enzyme kinetic analysis of Dop

Pupylation of 5-FAM-Lys resulting in Pup-(5-FAM-Lys) (Pup-Fl) and the fluorescence anisotropy-based depupylation experiments were carried out as described^19,22^. 0.005–0.15 µM ^Cglu^Dop (WT or DopGS variant) was incubated with 0.5 mM ATP at 30 °C for 5 min, and then the measurement was started by the addition of 0.0625–4 µM Pup-Fl that was also incubated in the same way as Dop in advance. The enzyme concentration was always kept at least 10-fold lower than the concentration of Pup-Fl. All the measurements were carried out in a plate reader (Synergy^TM^ 2, BioTek Instruments, Inc.) with the temperature set at 30 °C, the excitation or emission wavelength set to 485/20 nm or 528/20 nm, respectively. The initial velocity of depupylation of Pup-Fl by ^Cglu^DopWT or ^Cglu^DopGS variant was obtained by using an equation defined in this reference^39^,

1 $${\nu }_{0}=\frac{{r}_{0}-{r}_{t}}{{r}_{0}-{r}_{f}}\cdot \frac{{A}_{0}}{\triangle t}$$

where *ν*_0_ is the initial velocity, *r*_0_ is the anisotropy of Pup-Fl before adding Dop, *r*_*t*_ is the anisotropy after reaction for time *t*, *r*_*f*_ is the anisotropy after Pup-Fl is completely converted to Pup, and *A*_0_ is the concentration of Pup-Fl at the start of the reaction. *K*_*m*_ and *k*_cat_ were acquired by fitting the data to the Michaelis-Menten equation,

2 $$\frac{{\nu }_{0}}{{E}_{0}}=\frac{{k}_{{cat}}\cdot\, [{S}_{0}]}{\left[{S}_{0}\right]+{K}_{m}}$$

where [*S*_0_] and *E*_0_ denote the concentrations of Pup-Fl and Dop, respectively. The data was analyzed and plotted with GraphPad Prism 8.

### Observation of the depupylation lag phase of Pup-Fl

1.25 µM ^Cglu^Dop (WT or DopGS variant) was incubated with 5 μM Pup-Fl at 30 °C for 5 min, and then the measurement was started by addition of 100 µM ATP or ADP + 10 mM Na/K Pi (pH 8.0 at 23 °C). The excitation wavelength was set to 485/20 nm, and the emission wavelength was set to 528/20 nm using a plate reader (Synergy^TM^ 2, BioTek Instruments, Inc.). The plots were illustrated with GraphPad Prism 8.

### Protein crystallization

Crystallizations of Dop (*Acidothermus cellulolyticus*) alone or Dop-PupE/Q complexes (molar ratio = 1:2) in the presence of nucleotide were carried out in sitting drop vapor diffusion plates at a protein concentration of 6 mg/mL at 20 °C by mixing 1 µL of protein solution with 1 µL of reservoir solution. Dop with the Dop-loop inserted in the active site formed crystals in reservoir solutions consisting of 14–20 % (w/v) PEG 3350, 100 mM Bis-Tris propane pH 6.0–6.5 at 20 °C and 100–200 mM potassium thiocyanate (KSCN). Before flash freezing with liquid nitrogen, the crystals were briefly soaked with cryo-protectant buffer containing the crystallization buffer plus 30 % (w/v) PEG 400. Dop-PupQ-AMP-PCP and Dop-PupE^P^-ADP complexes formed crystals in reservoir solutions consisting of 100 mM Tris-acetate pH 7.0–8.5, 10 mM AMP-PCP or ADP, 40 mM MgCl_2_, and 0.75–1.0 M KH_2_PO_4_ at 20 °C. Before flash freezing with liquid nitrogen, the crystals of Dop-PupQ-AMP-PCP complex were soaked with cryo-protectant buffer (100 mM Tris-acetate pH 8.5, 35 % (w/v) PEG 400, 40 mM MgCl_2_ and 10 mM AMP-PCP) for 2 h at 20 °C. The crystals of Dop-PupE^P^-ADP complexes were soaked with the cryo-protectant buffer (100 mM Tris-acetate pH 7.5, 150 mM KCl, 30 % (w/v) PEG 400, 20 mM MgCl_2_, 5 mM ATP and 100 uM PupE) at 20 °C for 19 h. Dop-PupE-ADP-MgF_3_(H_2_O)^−^ complex formed crystals in reservoir solutions consisting of 100 mM Tris-acetate pH 7.0–8.5, 10 mM ADP, 40 mM MgCl_2_, 16 mM NaF, 4 mM BeSO_4_ and 0.75-1.0 M KH_2_PO_4_ at 20 °C. Before flash freezing with liquid nitrogen, the crystals were soaked with the cryo-protectant buffer (100 mM Tris-acetate pH 8.5, 35 % (w/v) PEG 400, 40 mM MgCl_2_, 16 mM NaF, 4 mM BeSO_4_ and 10 mM ADP) for 2 h at 20 °C. Dop-PupE-ADP-MgF_4_^2−^ complex formed crystals in reservoir solutions consisting of 100 mM Tris-acetate pH 7.0-8.5, 10 mM ADP, 100 mM MgCl_2_, 10 mM NaF and 0.75-1.0 M KH_2_PO_4_ at 20 °C. Before flash freezing with liquid nitrogen, the crystals were soaked with the cryo-protectant buffer (100 mM Tris-acetate pH 8.5, 35 % (w/v) PEG 400, 200 mM MgSO_4_, 16 mM NaF and 10 mM ADP) for 2 h at 20 °C.

### Data collection, structure determination, model building, and refinement

All X-ray diffraction data sets were collected at beamline X06SA of the Swiss Light Source (Paul Scherrer Institute, Villigen, Switzerland) and were indexed and integrated using XDS^40^. Data merging and scaling were carried out using the program AIMLESS^41^ from the CCP4 suite^42^. All structures were solved by molecular replacement with the program Phaser using the previously solved Dop structure (PDB 5LRT) as a search model. All atomic models were further improved by iterative model building in COOT^43^ and refinement in Phenix.refine^44^. Statistics are summarized in Table 1. All structure images were generated with PyMol (The PyMol Molecular Graphics System, version 2.0, Schrodinger, LLC).

### Structural comparison

Comparisons of structures in this study were performed by using the super or align command in PyMol depending on the similarities between the structures (The PyMol Molecular Graphics System, version 2.0, Schrodinger, LLC).

### Reporting summary

Further information on research design is available in the Nature Research Reporting Summary linked to this article.

## Supplementary information

Supplementary Information

Reporting summary

## Data Availability

Structure factors and atomic coordinates for structures of Dop-PupQ-AMP-PCP, Dop-PupE-ADP-MgF_3_(H_2_O)^−^, Dop-PupE^P^-ADP, Dop-PupE-ADP-MgF_4_^2−^ and Dop (Dop-loop-inserted) have been deposited in the Protein Data Bank under the accession codes 7OXY, 7OYF, 7OY3, 7OYH and 7OXV, respectively. Source data are provided with this paper.

## Source data

Source data
