# Supplementary material for: Structures of prokaryotic ubiquitin-like protein Pup in complex with depupylase Dop reveal the mechanism of catalytic phosphate formation
Source: Nat Commun. 2021 Nov 17;12:6635. doi: 10.1038/s41467-021-26848-x (PMC8599861; doi:10.1038/s41467-021-26848-x)
Supplement: Supplementary file 1 — Supplementary Information [file 41467_2021_26848_MOESM1_ESM.pdf]

Supplementary Information for

Structures of prokaryotic ubiquitin-like protein Pup in complex with depupylase Dop reveal the mechanism of catalytic phosphate formation

Hengjun Cui[1], Andreas U. Müller[1], Marc Leibundgut[1], Jiawen Tian[1], Nenad Ban[1] & Eilika Weber-Ban[1]*

[1]ETH Zurich, Institute of Molecular Biology & Biophysics, CH-8093 Zurich, Switzerland

* To whom correspondence should be addressed. E-mail: eilika@mol.biol.ethz.ch

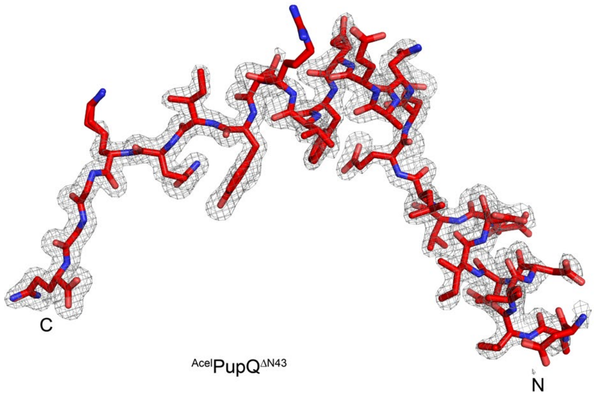

Supplementary Figure 1. ^{Acel}PupQ$^{\Delta N43}$ with simulated annealing polder map contoured at 2.5 σ. The simulated annealing polder map of the PupQ fragment in the AMP-PCP-bound Dop-PupQ complex structure was calculated using *phenix.refine* with the coordinates of the input model randomly displaced by 0.5 Å.

a

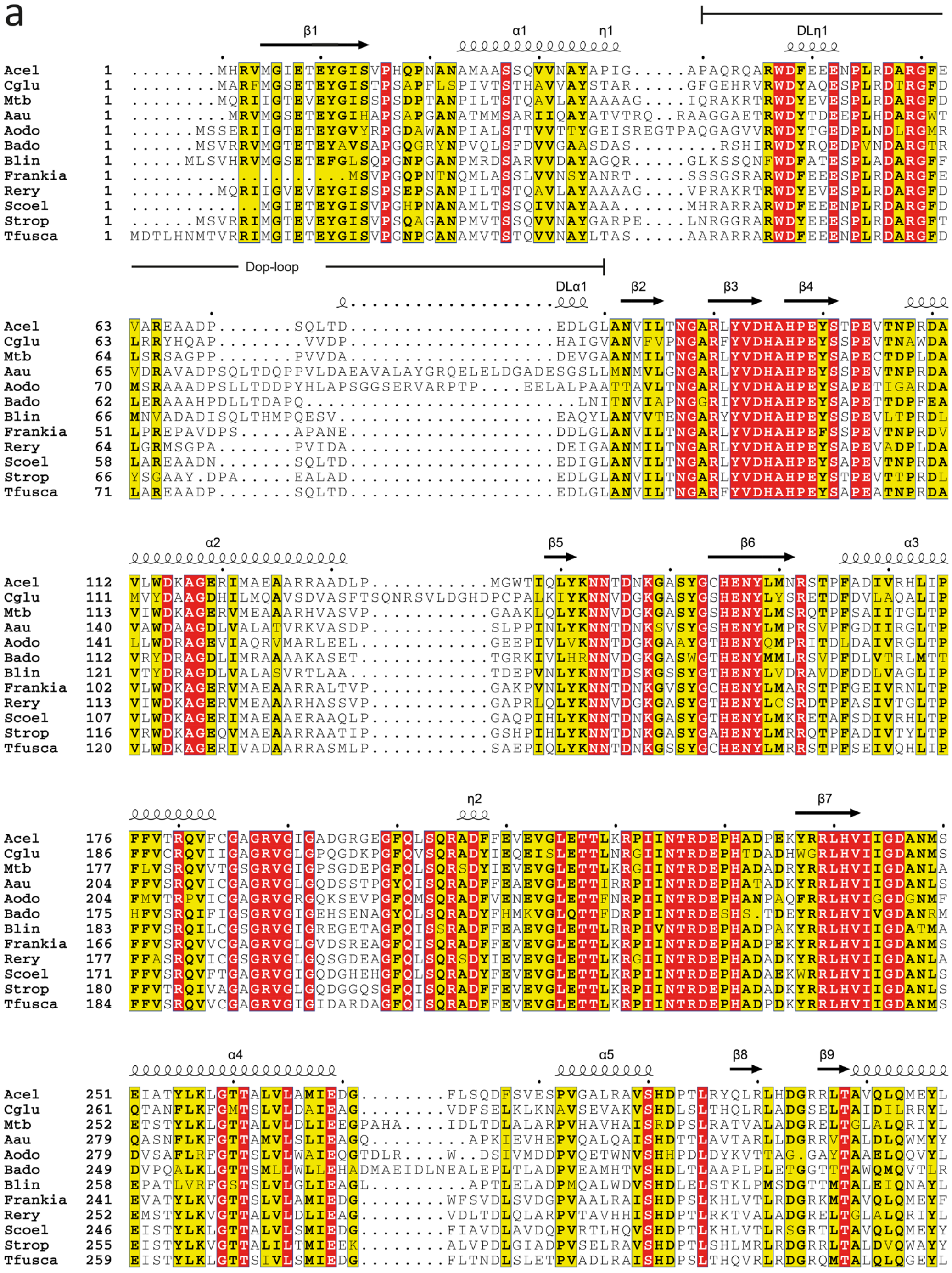

β1 α1 η1 DLη1
Acel 1 ........MHRVMGIETEYGISVPHQPNANAMAASSQVVNAYAPIG.....APAQRQARWDFEEENPLRDARGFE
Cglu 1 ........MARFMGSETEYGISTPSAPFLSPIVTSTHAVVAYSTAR.....GFGEHRVRWDYAQESPLRDTRGFD
Mtb 1 ........MQRIIGTEVEYGISSPSDPTANPILTSTQAVLAYAAAAG....IQRAKRTRWDYEVESPLRDARGFD
Aau 1 .........MRVMGSETEYGIHAPSAPGANATMMSARIIQAYATVTRQ..RAAGGAETRWDYTDEEPLHDARGWT
Aodo 1 .....MSSERIIGTETEYGVYRPGDAWANPIALSTTVVTTYGEISREGTPAQGAGVVRWDYTGEDPLNDLRGMR
Bado 1 .....MSVRRVMGTETEYAVSAPGQGRYNPVQLSFDVVGAASDAS........RSHIRWDYRQEDPVNDARGTR
Blin 1 .....MLSVHRVMGSETEFGLSQPGNPGANPMRDSARVVDAYAGQR.....GLKSSQNFWDFATESPLADARGFF
Frankia 1 .........................MSVPGQPNTNQMLASSLVVNSYANRT.....SSSGSRARWDFEEESPLRDARGFE
Rery 1 ........MQRIIGVEVEYGISSPSEPSANPILTSTQAVLAYAAAAG....VPRAKRTRWDYEVESPLRDARGFD
Scoel 1 ..........MGIETEYGISVPGHPNANAMLTSSQIVNAYAAA.....MHRARRARWDFEEENPLRDARGFD
Strop 1 .....MSVRRIMGTETEVRGISVPSQAGANPMVTSSQVVNAYGARPE....LNRGGRARWDYEEESPLRDARGFT
Tfusca 1 MDTLHNMTVRRIMGIETEYGISVPGNPGANAMVTSTQVVNAYLTAS.....AARARRARWDFEEENPLRDARGFD
Dop-loop
DLα1 β2 β3 β4
Acel 63 VAREAADP.......SQLTD..................EDLGLANVILTNGARLYVDHAHPEYSTPEVTNPRDA
Cglu 63 LRRYHQAP.......VVDP...................HAIGVANVFVPNGARFYVDHAHPEYSSPEVTNAWDA
Mtb 64 LSRSAGPP.......PVVDA..................DEVGAANMILTNGARLYVDHAHPEYSAPECTDPLDA
Aau 65 VDRAVADPSQLTDQPPVLDAEAVALAYGRQELELDGADESGSLLMNMVLGNGARLYVDHAHPEYSSPEVTNPRDA
Aodo 70 MSRAAADPSLLTDDPYHLAPSGGSERVARPTP....EELALPAATTAVLTNGARLYVDHAHPEYSAPETIGARDA
Bado 62 LERAAAHPDLLTDAPQ........................LNITNVIAPNGGRIYVDHAHPEYSAPETTDPFEA
Blin 66 MNVADADISQLTHMPQESV...................EAQYLANVVTENGARYYVDHAHPEYSSPEVLTPRDL
Frankia 51 LPREPAVDPS.....APANE..................DDLGLANVILTNGARLYVDHAHPEFSSPEVTNPRDV
Rery 64 LGRMSGPA.......PVIDA..................DEIGAANMILTNGARLYVDHAHPEYSAPEVADPLDA
Scoel 58 LAREAADN.......SQLTD..................EDIGLANVILTNGARLYVDHAHPEYSAPEVTNPRDA
Strop 66 YSGAAY.DPA.....EALAD..................EDLGLANVILTNGARLYVDHAHPEYSTPEVTTPRDL
Tfusca 71 LAREAADP.......SQLTD..................EDLGLANVILTNCARFYVDHAHPEYSAPEATNPRDA
α2 β5 β6 α3
Acel 112 VLWDKAGERIMAEAARRAADLP...........MGWTIQLYKNNTDNKGASYGCHENYLMNRSTPFADIVRHLIP
Cglu 111 MVYDAAGDHILMQAVSDVASFTSQNRSVLDGHDPCPALKIYKNNVDGKGASYGSHENYLYSRETDFDVLAQALIP
Mtb 113 VIWDKAGERVMEAAARHVASVP..........GAAKLQLYKNNVDGKGASYGSHENYLMSRQTPFSAIITGLTP
Aau 140 VAWDAAGDLVLALATVRKVASDP..........SLPPINLYKNNTDNKSVSYGSHENYLMPRSVPFGDIIRGLTP
Aodo 141 LLWDRAGEVIAQRVMARLEEL...........GEEPIVLVKNNTDGKGAAYGTHENYQMPRITDLDAIVRGLTP
Bado 112 VRYDRAGDLIMRAAAAKASET...........TGRKIVLHRNNVDGKGASWGTHENYMMLRSVPFDLVTRLMTT
Blin 121 VTYDRAGDLVALASVRTLAA............TDEPVNLYKNNTDSKGSSYGTHENYLVDRAVDFDDLVAGLIP
Frankia 102 VLWDKAGERVMAEAARRALTVP..........GAKPVNLYKNNTDNKGVSYGCHENYLMARSTPFGEIVRNLTP
Rery 113 VIWDKAGERVMEAAARHASSVP..........GAPRLQLYKNNVDGKGASYGTHENYLCSRDTPFASIVTGLTP
Scoel 107 VLWDKAGERIMAEAAERAAQLP..........GAQPIHLYKNNTDNKGASYGTHENYLMKRETAFSDIVRHLTP
Strop 116 VRWDKAGEQVMAEAARRAATIP..........GSHPIHLYKNNTDNKGASYGAHENYLMRRQTPFADIVTYLTP
Tfusca 120 VLWDKAGERIVADAARRASMLP..........SAEPIQLYKNNTDNKGSSYGCHENYLMRRSTPFSEIVQHLIP
η2 β7
Acel 176 FFVTRQVFCGAGRVGIGADGRGEGFQLSQRADFFEVEVGLETTLKRPIINTRDEPHADPEKYRRLHVIIGDANMS
Cglu 186 FFVCRQVIIGAGRVGLGPQGDKPGFQVSQRADYIEQEISLETTLNRGIINTRDEPHTDADHWGRLHVIIGDANMS
Mtb 177 FLVSRQVVTGSGRVGIGPSGDEPGFQLSQRSDYIEVEVGLETTLKRGIINTRDEPHADADRYRRLHVIIGDANLA
Aau 204 FFVSRQVICGAGRVGLGQDSSTPGYQISQRADFFEAEVGLETTIRRPIINTRDEPHATADKYRRLHVIIGDANLS
Aodo 204 FMVTRPVICGAGRVGRGQKSEVPGFQMSQRADFVENEVGLETTFNRPIINTRDEPHANPAQFRRLHVIGGDGNMF
Bado 175 HFVSRQIFIGSGRVGIGEHSENAGYQLSQRADYFHMKVGLQTTFDRPIINTRDESHS.TDEYRRLHVIVGDANRM
Blin 183 FFVSRQILCGSGRVGIGREGETAGFQISSRADFFEAEVGLETTLRRPIVNTRDEPHADPAKYRRLHVIIGDATMA
Frankia 166 FFVSRQVVCGAGRVGLGVDSREAGFQLSQRADFFEVEVGLETTLKRPIINTRDEPHADPEKYRRLHVIIGDANMS
Rery 177 FFASRQVICGSGRVGIGQSGDEAGFQLSQRSDYIEVEVGLETTLKRGIINTRDEPHADADKYRRLHVIIGDANLA
Scoel 171 FFVSRQVFTGAGRVGIGQDGHEHGFQLSQRADYFEVEVGLETTLKRPIINTRDEPHADAEKWRRLHVIIGDANLS
Strop 180 FFVTRQIVAGAGRVGLGQDGGQSGFQISQRADFFEVEVGLETTLKRPIINTRDEPHADADKYRRLHVIIGDANLS
Tfusca 184 FFVSRQVVCGAGRVGIGIDARDAGFQISQRADFFEVEVGLETTLKRPIINTRDEPHADPDKYRRLHVIIGDANMS
α4 α5 β8 β9
Acel 251 EIATYLKLGTTALVLAMIEDG........FLSQDFSVESPVGALRAVSHDPTLRYQLRLHDGRRLTAVQLQMEYL
Cglu 261 QTANFLKFGMTSLVLDAIEAG........VDFSELKLKNAVSEVAKVSHDLSLTHQLRLADGSELTAIDILRRYL
Mtb 252 ETSTYLKLGTTALVLDLIEEGPAHA....IDLTDLALARPVHAVHAISRDPSLRATVALADGRELTGLALQRIYL
Aau 279 QASNFLKFGTTAMVLSLIEAGQ.........APKIEVHEPVQALQAISHDTTLAVTARLLDGRRVTALDLQWMYY
Aodo 279 DVSAFLRFGTTSLVLWAIEQGTDLR....W..DSIVMDDPVQETWNVSHHPDLDYKVTTAG.GAYTAAELQQVYL
Bado 249 DVPQALKLGTTSMLLWLLEHADMAEIDLNEALEPLTLADPVEAMHTVSHDLTLAAPLPLETGGTTTAWQMQVTLR
Blin 258 EPATLVRFGSTSLVLGLIEAGL.........APTLELADPMQALWDVSHDLELSTKLPMSDGTTMTALEIQNAYL
Frankia 241 EVATYLKVGTTALVLAMIEDG........WFSVDLSVDGPVAALRAISHDPSLKHLVTLRDGRKMTAVQLQMEYF
Rery 252 EMSTYLKVGTTALVLDLIEAG........VDLTDLQLARPVTAVHHISHDPTLRKTVALADGRELTGLALQRIYL
Scoel 246 EISTYLKLGTTALVLSMIEDG........FIAVDLAVDQPVRTLHQVSHDPTLKHLVTLRSGRTLTAVQLQMEYY
Strop 255 EISTYLKVGTTALILTMIEEK........ALVPDLGIADPVSELRAVSHDPSLSHLMRLRDGRRLTALDVQWAYY
Tfusca 259 EISTYLKLGTTSIVLSMIEDG........FLTNDLSLETPVADLRAISHDPSLTHQVRLRDGRQMTALQLQGEYL

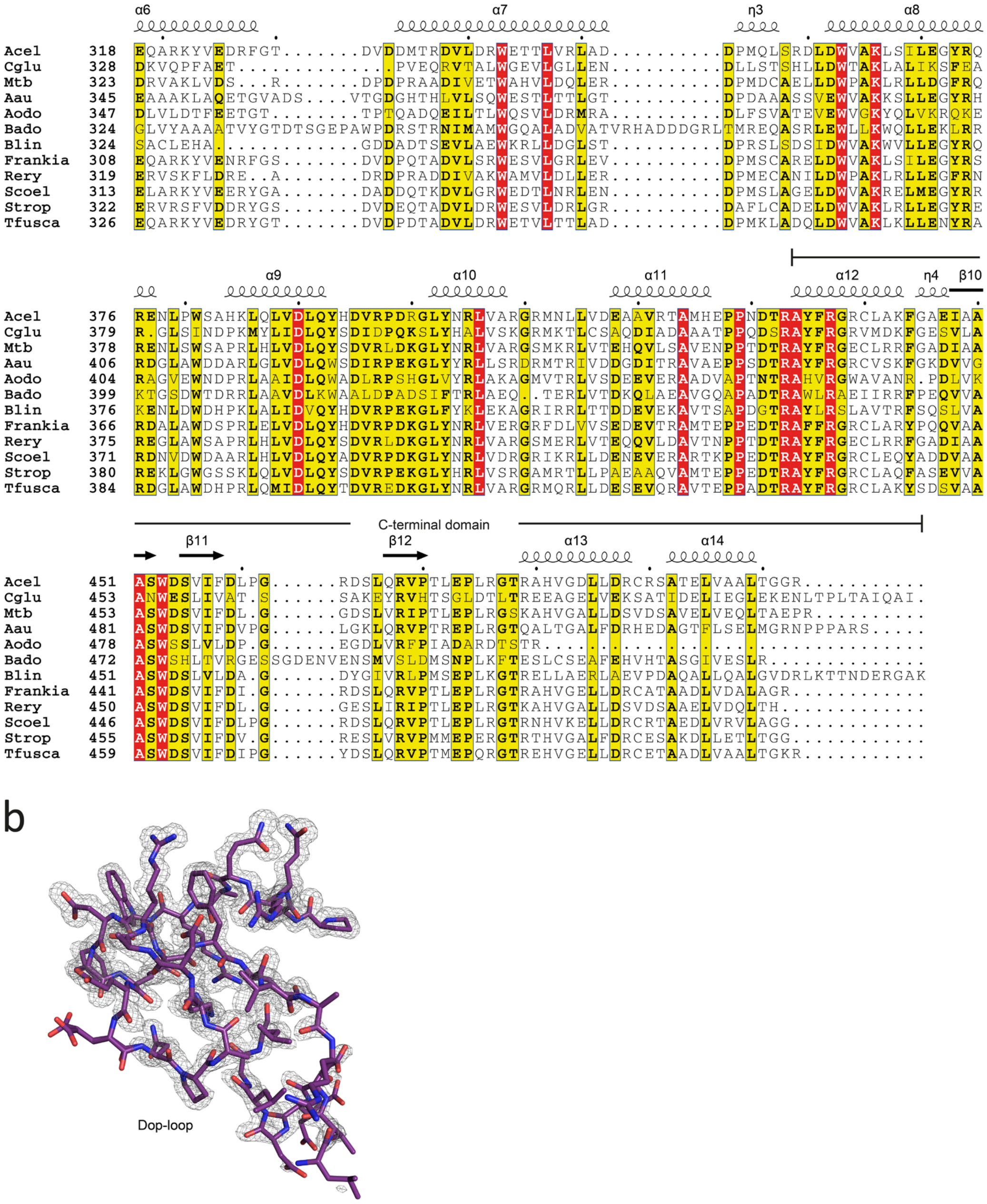

Supplementary Figure 2. Overview of Dop sequence and structural elements. a, Sequence alignment of Dop orthologs and linear depiction of secondary structure elements based on the structure of ^{Acel}Dop with the Dop-loop inserted into the active site. Dop homologs of the following

organisms were aligned. *Acidothermus cellulolyticus* (Acel), *Arthrobacter aurescens* (Aau), *Actinomyces odontolyticus* (Aodo), *Bifidobacterium adolescentis* (Bado), *Brevibacterium linens* (Blin), *Corynebacterium glutamicum* (Cglu), *Frankia sp.* (Frankia), *Mycobacterium tuberculosis* (Mtb), *Rhodococcus erythropolis* (Rery), *Streptomyces coelicolor* (Scoel), *Salinispora tropica* (Strop) and *Thermobifida fusca* (Tfusca). Secondary structure elements are indicated above the sequence alignment. Strictly conserved residues are shown as white letters with red background, and similar residues are shown as black letters with yellow background. α: α-helix; β: β-sheet; η: 3_{10} helix. This figure was generated by ESPript 3.0 (http://espript.ibcp.fr/ESPript/cgi-bin/ESPript.cgi). **b**, Stick representation of the Dop-loop as observed in the Dop-loop-inserted crystal structure. The simulated annealing polder map of the Dop-loop at 2.5 σ contour level was calculated using *phenix.refine* with the coordinates of the input model randomly displaced by 0.5 Å.

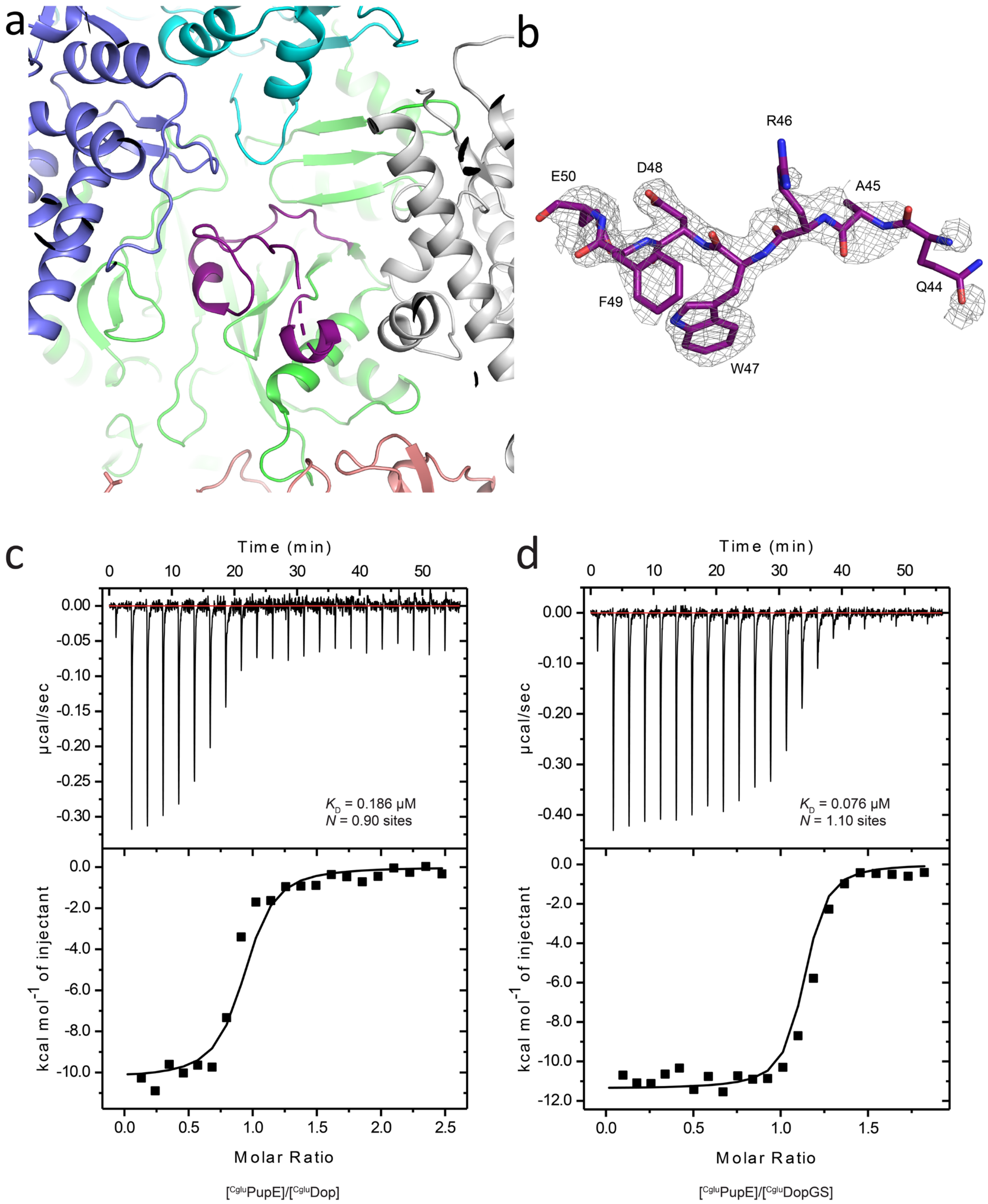

Supplementary Figure 3. a, Crystal contacts of the Dop-loop-inserted Dop crystal structure indicate that the Dop-loop conformation is not induced by crystal contacts. In the center one Dop molecule is shown in green with the Dop-loop depicted in purple. Four other Dop molecules involved in crystal contacts with the Dop molecule shown in green are depicted in blue, cyan, grey and salmon. The Dop-loop does not mediate any of the contacts and can freely move within the lattice. **b**, Stick representation of the partially-resolved Dop-loop in the Dop-PupE-ADP-$MgF_3(H_2O)^-$ complex structure with the simulated annealing polder map contoured at 3.0 σ. **c** and **d**, Measurement of binding affinities of ^{Cglu}PupE to ^{Cglu}DopWT or to the ^{Cglu}DopGS variant using isothermal titration calorimetry.

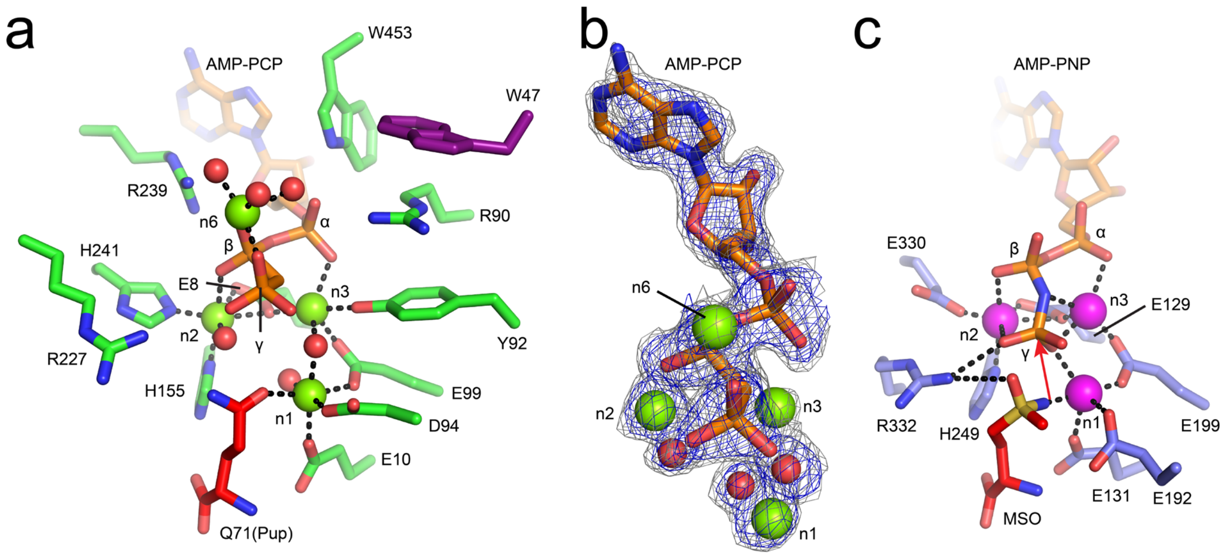

Supplementary Figure 4. AMP-PCP-bound Dop-PupQ complex structure represents the ground state of ATP hydrolysis. a, Close-up of the active site of the Dop-PupQ-AMP-PCP complex (**Fig. 1a**) with bound AMP-PCP (orange), magnesium ions (green spheres), water molecules (red spheres) and the C-terminal Pup residue (Q71, red). The residues of Dop are colored green except for residue W47 from the Dop-loop that is colored purple. Polar interactions are represented as black dashed lines. **b**, The unbiased mFo-DFc Fourier map of AMP-PCP, magnesium ions and water molecules at 2.0 σ (grey) or 3.0 σ (blue) contour level was calculated during molecular replacement with PDB code 5LRT (which did not include any ligands) followed by three rounds of refinement in Phenix without any further model building. **c**, Active site of glutamine synthetase (GS, PDB 2D3B) with bound AMP-PNP (orange), manganese ions (pink spheres) and methionine sulfoximine (MSO) shown in the same orientation as the Dop active site in **a,** which was omitted here for clarity. The red arrow in the figure indicates the nucleophilic attack initiated by MSO. The residues from GS were colored in blue. Polar interactions are represented as black dashed lines.

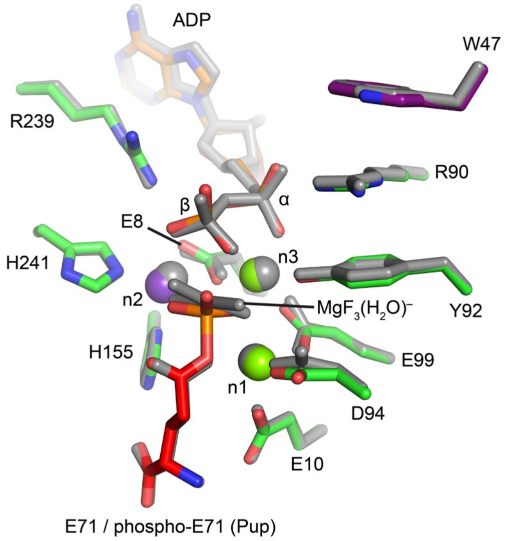

Supplementary Figure 5. Structural comparison between the Dop-PupE^{P}-ADP complex structure (green, red, orange and purple) and the Dop-PupE-ADP-$MgF_3(H_2O)^-$ complex structure (grey). Binding of K^+ at the n2 site does not affect the position of ADP or the C-terminal Pup residue, as indicated by the almost perfect overlay in both structures.

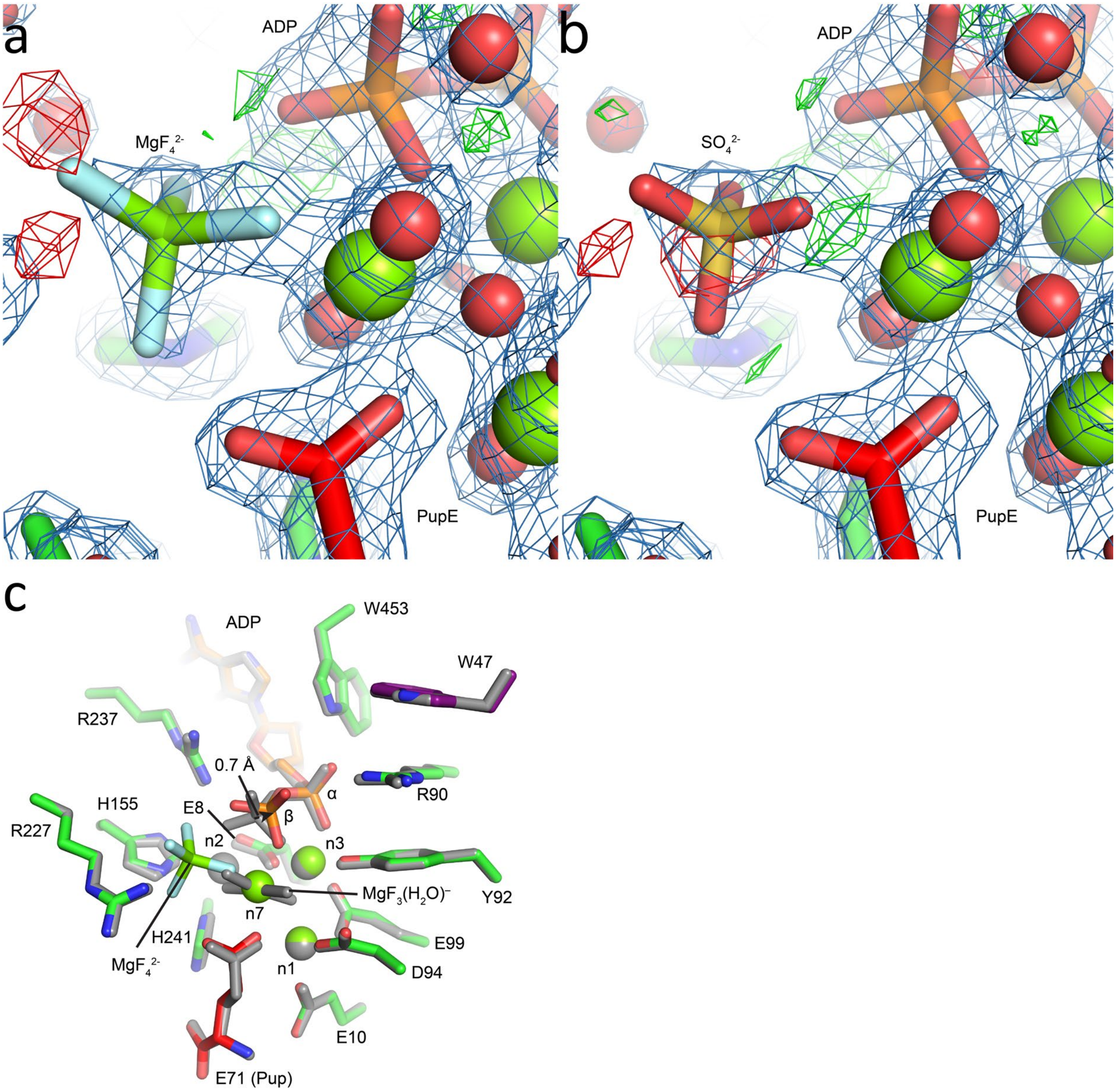

Supplementary Figure 6. a and **b,** Structure refinement of the Dop-PupE-ADP complex with either MgF_4^{2-} (**a**) or SO_4^{2-} (**b**) in the model against the same X-ray data set. 2mFo-DFc maps (blue) were contoured at 2.0 σ; mFo-DFc maps (green / red) were contoured at + / - 3.0 σ. **c**, Structural comparison between the Dop-PupE-ADP-MgF_4^2 complex structure (green, orange, red and purple) and the Dop-Pup-ADP-$MgF_3(H_2O)^–$ complex structure (grey). Magnesium bound at the n7 site in the former structure is located where the $MgF_3(H_2O)^–$ binds in the latter structure.

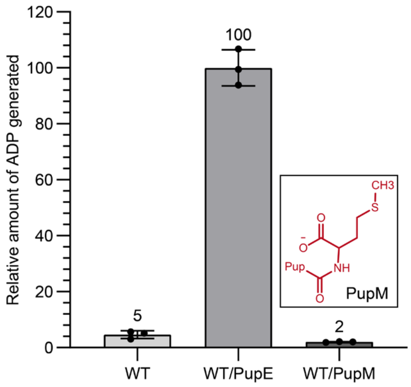

Supplementary Figure 7. ATP hydrolysis assays carried out by anion exchange chromatography. 30 µM ^{Cglu}Dop was incubated with or without 40 µM ^{Cglu}Pup (PupE or PupM) in the presence of 1 mM ATP at 30 °C for 15 min. The reaction was stopped by addition of 6 M Urea and then subject to analysis by Resource Q column (1 ml). Each reaction was carried out in three independent replicates and data are represented as mean value +/- SD.

Supplementary Table 1: Oligonucleotide primers used in this study. Mutations are shown in lowercase letters.

**Primer name**	**Sequence (5'→3')**	**Purpose**
CgluDopGS-fw	GATCGGGATCGGGTGTGGCCAACGTGTTTG	Amplification of DopGS variant from DopWT expression vector
CgluDopGS-rv	CCGATCCCGATCCCTCACCAAATCCACGCGCGG	See CgluDopGS-fw
CgluPupM-fw	GGCatgTAGGAGCTCCGTCG	Amplification of PupM variant from PupE expression vector
CgluPupM-rv	ACCCTTTTGTACATAGGAACGAACG	See CgluPupM-fw
