# Supplementary material for: Structures of prokaryotic ubiquitin-like protein Pup in complex with depupylase Dop reveal the mechanism of catalytic phosphate formation
Source: Nat Commun. 2021 Nov 17;12:6635. doi: 10.1038/s41467-021-26848-x (PMC8599861; doi:10.1038/s41467-021-26848-x)
Supplement: Supplementary file 2 — Reporting summary [file 41467_2021_26848_MOESM2_ESM.pdf]

nature research

Corresponding author(s): Eilika Weber-Ban

Last updated by author(s): Oct 20, 2021

Reporting Summary

Nature Research wishes to improve the reproducibility of the work that we publish. This form provides structure for consistency and transparency in reporting. For further information on Nature Research policies, see Authors & Referees and the Editorial Policy Checklist .

Statistics

For all statistical analyses, confirm that the following items are present in the figure legend, table legend, main text, or Methods section.

n/a	Confirmed	
☐	☒	The exact sample size (*n*) for each experimental group/condition, given as a discrete number and unit of measurement
☐	☒	A statement on whether measurements were taken from distinct samples or whether the same sample was measured repeatedly
☒	☐	The statistical test(s) used AND whether they are one- or two-sided<br>*Only common tests should be described solely by name; describe more complex techniques in the Methods section.*
☒	☐	A description of all covariates tested
☒	☐	A description of any assumptions or corrections, such as tests of normality and adjustment for multiple comparisons
☐	☒	A full description of the statistical parameters including central tendency (e.g. means) or other basic estimates (e.g. regression coefficient) AND variation (e.g. standard deviation) or associated estimates of uncertainty (e.g. confidence intervals)
☒	☐	For null hypothesis testing, the test statistic (e.g. *F*, *t*, *r*) with confidence intervals, effect sizes, degrees of freedom and *P* value noted<br>*Give P values as exact values whenever suitable.*
☒	☐	For Bayesian analysis, information on the choice of priors and Markov chain Monte Carlo settings
☒	☐	For hierarchical and complex designs, identification of the appropriate level for tests and full reporting of outcomes
☒	☐	Estimates of effect sizes (e.g. Cohen's *d*, Pearson's *r*), indicating how they were calculated

Our web collection on statistics for biologists contains articles on many of the points above.

Software and code

Policy information about availability of computer code

Data collection	Swiss Light Source (SLS, Paul-Scherrer-Institut, Villigen, Switzerland) in-house software for beamline operation
Data analysis	XDS (BUILT=20200417), AIMLESS 0.7.4, Phenix 1.18.2_3874, Phaser 2.8.3, Coot 0.9.4, PyMOL 2.0, ESPript 3.0, GraphPad Prism 8, ChemDraw Professional 16, Origin 7.0, GelAnalyzer software (version 2010a)

For manuscripts utilizing custom algorithms or software that are central to the research but not yet described in published literature, software must be made available to editors/reviewers. We strongly encourage code deposition in a community repository (e.g. GitHub). See the Nature Research guidelines for submitting code & software for further information.

Data

Policy information about availability of data

All manuscripts must include a data availability statement. This statement should provide the following information, where applicable:

- Accession codes, unique identifiers, or web links for publicly available datasets
- A list of figures that have associated raw data
- A description of any restrictions on data availability

Structural data are deposited in the protein data bank (PDB 7OXY, 7OYF, 7OY3, 7OYH and 7OXV). Other datasets are either contained in the main manuscript or provided as supplementary or source data files. Other data are available from the corresponding author upon reasonable request.

Field-specific reporting

Please select the one below that is the best fit for your research. If you are not sure, read the appropriate sections before making your selection.

Life sciences study design

All studies must disclose on these points even when the disclosure is negative.

Sample size	No statistical methods were used to predetermine sample sizes. Required experimental sample sizes were chosen according to common practice in enzymology (three or two independent experiments, as indicated in the figure legends). Statistical analysis was limited to determine mean ± SD.
Data exclusions	Data exclusion for X-ray analysis was based on CC1/2 > 0.6
Replication	Experiments were repeated individually three or more times. Results could be reproduced successfully each time.
Randomization	Randomization is not applicable in our study, as X-ray crystallography has internal measures (Rfree) to avoid bias.
Blinding	Our study did not involve experiments where blinding was required or appropriate. None of the experiments involved human or animal models or group allocation.

Reporting for specific materials, systems and methods

We require information from authors about some types of materials, experimental systems and methods used in many studies. Here, indicate whether each material, system or method listed is relevant to your study. If you are not sure if a list item applies to your research, read the appropriate section before selecting a response.

Materials & experimental systems

n/a	Involved in the study
☒	☐ Antibodies
☒	☐ Eukaryotic cell lines
☒	☐ Palaeontology
☒	☐ Animals and other organisms
☒	☐ Human research participants
☒	☐ Clinical data

Methods

n/a	Involved in the study
☒	☐ ChIP-seq
☒	☐ Flow cytometry
☒	☐ MRI-based neuroimaging
